# Dual Interlocked Mediators Enable Single-Ion-Conducting Quasi-Solid-State Electrolytes for Ultrafast-Charging Long-Life Sodium Metal Batteries

**DOI:** 10.1007/s40820-026-02236-2

**Published:** 2026-05-21

**Authors:** Yuan Zhang, Long Pan, Cheong Wa Leong, Xing-Guo Qi, Xiaozhong Huang, Xinyi Cai, Mufan Cao, Min Gao, Haoyu Zhang, Dawei Sha, Yang Zhou, ZhengMing Sun

**Affiliations:** 1 State Key Laboratory of Engineering Materials for Major Infrastructure, School of Materials Science and Engineering, Southeast University, Nanjing, 211189 People’s Republic of China; 2 HiNa Battery Technology Co., Ltd., Beijing, 213300 People’s Republic of China; 3 Institute of Technology for Carbon Neutralization, College of Electrical, Energy and Power Engineering, Yangzhou University, Yangzhou, 225009 People’s Republic of China

**Keywords:** Interlocked mediator engineering, Single-ion-conduction, Quasi-solid-state electrolyte, Electrode–electrolyte interphase, Sodium metal batteries

## Abstract

Quasi-solid-state electrolytes (QSEs) are critical for ultrafast-charging yet high-safety sodium metal batteries (SMBs), yet their implementation is hindered by sluggish Na^+^ transport in bulk and at interfaces. Here, we propose dual interlocked mediator engineering that transcends conventional independent approaches by coupling cationic Sn^2+^ salt with anionic difluoro(oxalato)borate (DFOB⁻) salts to simultaneously regulating bulk ion transport and bilateral interface chemistry. During QSE preparation, Sn^2+^ initiates in situ cationic polymerization, while DFOB⁻ acts as a retarding agent to suppress runaway polymerization. The first interlocking effect in the Sn-FB QSE bulk builds a uniform network, enabling near-unity Na^+^ transference number (0.94) and robust puncture strength (8.5 kPa). During cell operation, Sn^2+^ is reduced to form a hybrid NaSn alloy-based solid-electrolyte interphase, while DFOB⁻ oxidizes to generate a robust yet thin cathode–electrolyte interphase, respectively. This second interlocking effect creates adaptable bilateral interphases that facilitate Na^+^ diffusion and mitigate interfacial degradation. As a result, the symmetric cells exhibit 6000 h stability, and full cells retain 80.1 mAh g^–1^ at an ultrafast-charging rate of 15C and retain 90% capacity at 3C over 2000 cycles. Furthermore, high-mass-loading full cells and pressure-free pouch cells are demonstrated, underscoring the potential of dual interlocked mediator engineering for practical SMBs.

**Supplementary Information:**

The online version contains supplementary material available at 10.1007/s40820-026-02236-2.

## Highlights

- Single-ion conduction. Sn-FB quasi-solid-state electrolytes (QSEs) achieves near-unity Na^+^ transference number (0.94) and high conductivity (1.3 mS cm^–1^) via synergistic Sn^2+^ initiated polymerization and difluoro(oxalato)borate (DFOB⁻) retardation.
- Highly adaptable bilateral interphases. Sn^2+^ was reduced to form a hybrid NaSn alloy/inorganic-rich solid-electrolyte interphase to homogenize electric fields, while DFOB^–^ was oxidized to form robust, yet thin cathode–electrolyte interphase for rapid interfacial kinetics.
- Superior electrochemical performances. Sn-FB QSE delivers 6000 h stability without dendrite and retains 90% capacity after 2000 cycles at 3C.

**Supplementary Information:**

The online version contains supplementary material available at 10.1007/s40820-026-02236-2.

## Graphical abstract

**Figure:**
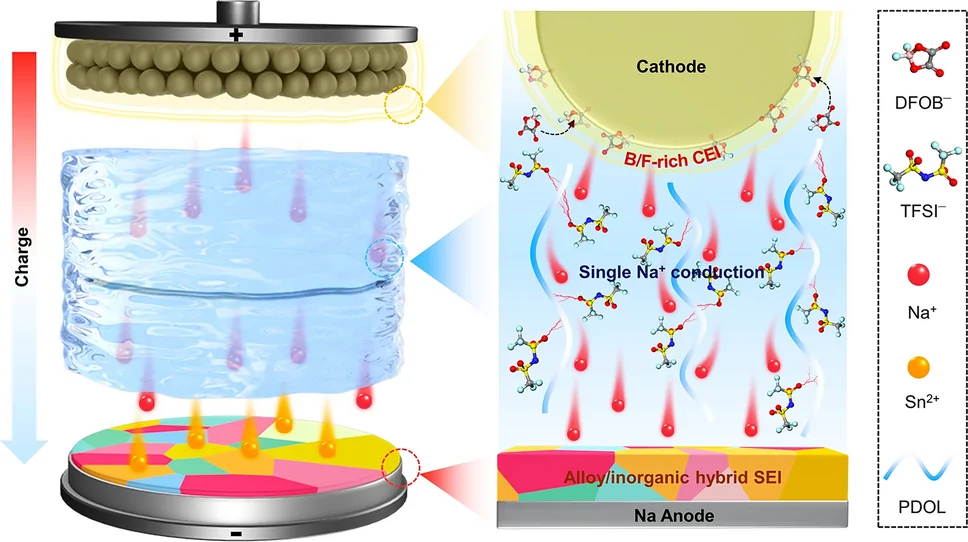

## Introduction

Ultrafast-charging sodium metal batteries (SMBs) are attracting intensive interest as a cost-effective alternative to Li systems, leveraging earth-abundant Na resources and a supply chain less exposed to price volatility [1–5]. Realizing ultrafast charge (≥ 3C), however, hinges on electrolytes that concurrently provide high Na conductivity (*σ*) and Na^+^ transference number (${t}_{{Na}^{+}}$) [6, 7]. Although conventional liquid electrolytes are effective, they amplify safety risks and promote inhomogeneous Na^+^ flux, aggravating dendritic failure, thus motivating quasi-solid-state electrolyte (QSE) designs that retain excellent interfacial wetting and scalability yet enhance intrinsic safety [8]. Despite these advantages, the reported state-of-the-art QSEs remain two challenges under ultrafast-charging conditions [9–12]. (i) Limited ion transport in bulk: the strong Na⁺-polymer coordination and anion-dominated conduction depress ${t}_{{Na}^{+}}$ and slow down bulk Na^+^ migration. (ii) Poor ion diffusion at bilateral interfaces: the compositionally mismatched solid-electrolyte interphase (SEI) and cathode–electrolyte interphase (CEI) layers fail to accommodate high Na⁺ fluxes, leading to heterogeneous electric fields, dendrite formation, and accelerated QSE degradation. Therefore, addressing these coupled Na^+^ transport in bulk and at bilateral interfaces challenges is pivotal to unlocking practical ultrafast-charging SMBs.

To meet the stringent ionic transport in bulk demands of ultrafast-charging, elevating the ${t}_{{\mathrm{Na}}^{+}}$ from anion-dominated regimes toward single-ion conduction is essential [13–15]. However, the current state-of-the-art QSEs still exhibit a relatively low ${t}_{{Na}^{+}}$ (0.4–0.7), indicating substantial anion participation and concentration polarization, which intrinsically limits their application for ultrafast-charging SMBs. A mainstream route to raise ${t}_{{\mathrm{Na}}^{+}}$ and obtain a single-ion-conducting QSE is to immobilize anions via polymer-chain functionalization (e.g., tethering anionic groups or constructing polymer backbones) [16]. While conceptually effective, these modified polymers are cumbersome to obtain, and even depress *σ* by intentionally strengthening Na⁺-polymer coordination and increasing segmental friction, thereby trading diminished *σ* and rate performance for ${t}_{{\mathrm{Na}}^{+}}$ gains [17]. Consequently, there remain determined requirements for rational QSEs designs that achieve near-unity ${t}_{{\mathrm{Na}}^{+}}$ without complex polymer modifications, preserving high *σ* while enabling robust, ultrafast-charging-capable ${t}_{{\mathrm{Na}}^{+}}$ through the bulk.

Beyond Na^+^ transport in bulk, QSE–electrode interfacial failures under ultrafast-charging conditions remain another decisive barrier derived from interfacial ion diffusion hindrance [18–20]. At the anode side under high Na⁺ flux conditions, sodiophilic alloying effectively lowers the nucleation barrier but fails to suppress long-term morphological instability or maintain interfacial integrity under repeated plating/stripping [21, 22]. Conversely, introducing an inorganic-rich SEI provides higher modulus and favorable ion transport pathways, yet without improved nucleation, it still suffers from heterogeneous deposition and void formation [12]. These complementary limitations motivate a synergistic SEI strategy that couples sodiophilic alloying with inorganic-rich components, in which the former homogenizes initial Na nucleation, while the latter sustains uniform electric fields and rapid Na^+^ transport throughout cycling, jointly stabilizing plating/stripping under ultrafast-charging conditions [23–25]. Equally critical is the cathode interface, where high potentials and large current densities trigger oxidative side reactions. Specifically, fragile and nonuniform CEI formation and degradation of the electrolyte matrix collectively restrict Na^+^ transport at both the interface and the bulk [26–28]. A frontier-orbital guided mediator selection offers a unified handle at both electrodes: a mediator with a lower lowest unoccupied molecular orbital (LUMO) energy level is preferentially reduced at the Na surface, seeding a hybrid NaSn alloy/inorganic-rich SEI that equalizes local fields; in parallel, a mediator with a higher highest occupied molecular orbital (HOMO) energy level oxidizes first at the cathode to build a thin, tough, and inorganic-rich CEI, which can suppress electrolyte degradation while maintaining rapid Na⁺ transport [29, 30]. However, integrating bulk single-ion conduction with highly adaptable SEI and CEI layers under ultrafast-charging conditions remains unsolved [31].

Herein, transcending conventional independent approaches, a dual interlocked mediator engineering is proposed to prepare an advanced QSE (labeled as Sn-FB QSE) featuring both single-ion-conducting properties in bulk and highly adaptable interphases at the bilateral electrolyte–electrode interfaces, brought by dual interlocking effects. Specifically, the interlocked mediators are composed of a low-LUMO-energy-level cationic Sn^2+^-containing salt and a high-HOMO-energy-level anionic difluoro(oxalato)borate (DFOB^–^) salt. During Sn-FB QSE preparation, Sn^2+^ initiates the in situ cationic polymerization of 1,3-dioxolane (PDOL), while DFOB^–^ functions as a polymerization retarder to suppress runaway polymerization. This first interlocking effect in bulk yields a uniform, mechanically reinforced network with single-ion-conducting capability (0.94), excellent Na^+^ conductivity (1.3 mS cm^–1^), and puncture strength of 8.5 kPa. During cell operation, the second interlocking effect between Sn-FB QSE and bilateral electrodes creates adaptable interphases. Specifically, Sn^2+^, along with other electrolyte components, is preferentially reduced at the anode to form a hybrid SEI layer containing NaSn alloys and inorganic-rich species, which homogenizes the electric field. Meanwhile, DFOB^–^ sacrificially oxidizes at the cathode to form a thin yet robust CEI, mitigating electrolyte degradation and lowering Na^+^ diffusion resistance. The dual interlocking effects enable Sn-FB QSE with stable dendrite-free Na^+^ plating/stripping for 6000 h in symmetric cells [32]. Coupled with Na_3_V_2_(PO_4_)_3_ (NVP) cathode, full cells retain 90% capacity over 2000 cycles at 3C and deliver 80.1 mAh g^–1^ at an ultrafast rate of 15C. In addition, high-mass-loading full cells and pressure-free pouch cells are demonstrated. The superiority of dual interlocked mediator engineering strategy underscores its potential for practical ultrafast-charging and long-life SMBs.

## Experimental Section

### Synthesis of FB LE, Sn QSE, and Sn-FB QSE

To synthesize a liquid sample (FB LE), 1.5 g of sodium bis(trifluoromethylsulfonyl)imide (NaTFSI) and 0.1 g of sodium difluoro(oxalato)borate (NaDFOB) were dissolved in 10 mL of 1,3-dioxolane (DOL) in turn, followed by 1.0 mL of fluoroethylene carbonate (FEC) injected. On this base, a quasi-solid-state sample (Sn-FB QSE) was synthesized by in situ polymerization of LE with tin(II) trifluoromethanesulfonate (Sn(OTf)_2_) as the Lewis acid initiator while Sn QSE was polymerized without NaDFOB. All samples were kept at 25 °C for 48 h to ensure complete polymerization.

## Results and Discussion

### Design and Structural Characterizations

**Fig. 1:**
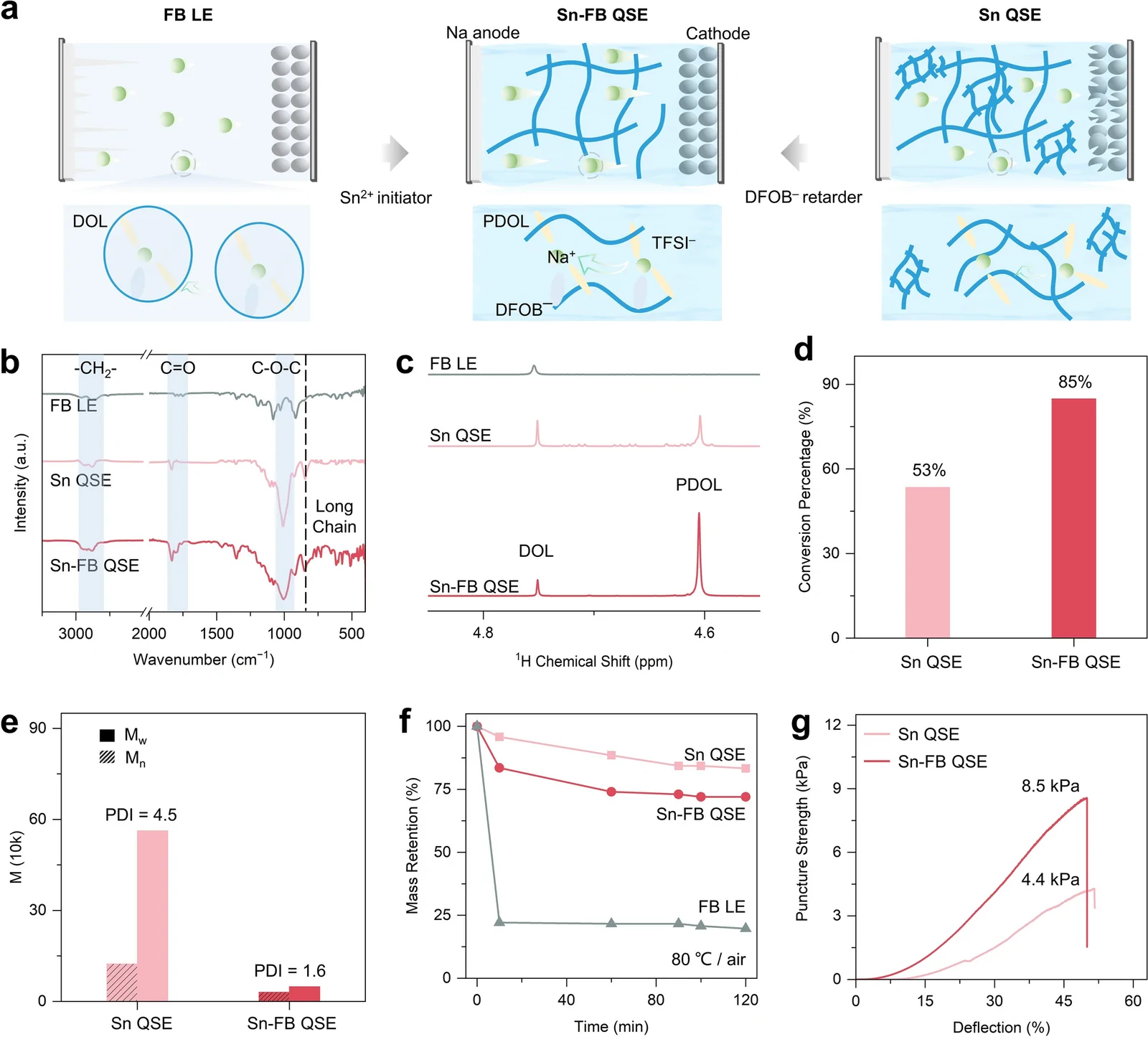
Structural and mechanical characterizations. **a** Synthesis process illustration of FB LE, Sn QSE, and Sn-FB QSE. **b** FT-IR spectra of FB LE, Sn QSE, and Sn-FB QSE. **c** ^1^H NMR spectra of FB LE, Sn QSE, and Sn-FB QSE, as well as **d** corresponding PDOL conversion percentage. **e** M_w_ and M_n_ of Sn QSE and Sn-FB QSE. **f** Mass retention at 80 °C in air of FB LE, Sn QSE, and Sn-FB QSE. **g** Puncture strength of Sn QSE and Sn-FB QSE

As illustrated in Fig. 1a, the Sn-FB QSE was synthesized via in situ polymerization of DOL, coupling Sn(OTf)_2_ as the Lewis acid initiator (Fig. S1a, b), and NaDFOB as the runaway polymerization retarder [33, 34]. For comparison, FB LE was synthesized without Sn(OTf)_2_, and Sn QSE was synthesized without NaDFOB. Vividly, a uniform polymerization product is presented in Sn-FB QSE. In contrast, FB LE presents its liquidity due to the lack of Sn^2+^ while Sn QSE presents its ununiform product after runaway polymerization due to the lack DFOB^–^ and brings the uneven molecular weight distribution of PDOL. Electrostatic potential (ESP) distribution was applied to validate DFOB^–^ works as polymerization retarder (Fig. S1c). Upon coordination with DFOB⁻, the blue coloration surrounding the Sn^2+^ center diminishes significantly, while the negative potential (yellow regions) of DFOB⁻ shifts toward Sn^2+^. This visualizes the electron transfer occurs from the oxalate and fluorine atoms toward the Sn center, effectively dispersing the excess positive charge of the Sn^2+^ and thereby reducing its electrophilicity. Subsequently, 50 μL of the precursor solution for each sample was injected into the prepared cells and the obtained composite Sn-FB QSE/separator was only 27 μm (Fig. S2).

The structures of FB LE, Sn QSE, and Sn-FB QSE were characterized by Fourier transform infrared (FT-IR) spectroscopy, as shown in Fig. 1b. The adsorption peak at around 2800 cm^–1^ corresponds to –CH_2_– stretching vibration segment, which also exists in pure DOL (Fig. S3) [35]. In addition, adsorption peaks near 1800 and 1000 cm^–1^ are assigned to C=O and C–O–C stretching vibration segments, respectively, which are attributed to the incorporation of NaDFOB and ether. Notably, the adsorption peak at ~ 920 cm^–1^ represents the long chain in both Sn QSE and Sn-FB QSE, corresponding to the successful incorporation of PDOL [36]. In order to further evaluate the conversion percentage of DOL into PDOL, ^1^H nuclear magnetic resonance (NMR) spectroscopy was conducted on all three samples. As shown in Fig. 1c, a chemical shift at 4.75 ppm corresponds to unconverted DOL, while the new signal at 4.61 ppm observed only in Sn QSE and Sn-FB QSE is attributed to PDOL [37]. Based on the integration of each peak, the conversion percentage in Sn-FB QSE is calculated as 85%, significantly higher than that in Sn QSE (53%) in Fig. 1d.

In addition, the mass-average molecular weight (M_w_) and the number-average molecular weight (M_n_) of Sn QSE and Sn-FB QSE were determined using gel permeation chromatography (GPC) and listed in Table S2. As visualized in Fig. 1e, the M_w_ and M_n_ of Sn QSE were calculated to be 562,581 and 123,699, respectively, while those of Sn-FB QSE were 48,783 and 30,634. Furthermore, to evaluate the discrepancy in dispersity between obtained QSEs, polydispersity index (PDI) was found to be 4.5 for Sn QSE and 1.6 for Sn-FB QSE (calculated based on M_w_/M_n_). The significantly higher PDI in Sn QSE indicates a broader and more uneven molecular weight distribution of PDOL, consistent with the runaway polymerization and low conversion percentage, indicating the role of NaDFOB as a polymer retarder [38, 39]. High-molecular-weight chains in Sn QSE are thermodynamically prone to regular folding and arrangement, leading to increased crystallinity as evidenced by the XRD patterns (Fig. S1d), Sn QSE exhibits a sharp diffraction peak at approximately 20°, confirming a high degree of crystallinity, whereas Sn-FB QSE displays a typical amorphous state. In polymer electrolytes, Na^+^ transport relies heavily on the segmental motion of polymer chains within amorphous regions. The crystalline domains in Sn QSE effectively lock the polymer segments into a rigid lattice, severely hindering their mobility and increasing the energy barrier for Na^+^ migration. Consequently, the ionic conductivity of Sn QSE is significantly lower than that of the amorphous Sn-FB QSE. Conducted at 80 °C in air (Fig. 1f), the mass retention of FB LE decreased rapidly to merely 20% within 12 min and remained at that level till 120 min, corresponding to the rapid evaporation of solvent. In contrast, both Sn QSE and Sn-FB QSE exhibited significantly higher mass retention of 83% and 72% after 120 min, respectively, showing the enhanced thermal stability as a result of polymerization.

The more homogenous polymerization also contributes to a more robust structure in Sn-FB QSE. Therefore, puncture strength–deflection curves for both Sn QSE and Sn-FB QSE were recorded (Fig. 1g). The puncture strength in Sn-FB QSE reached approximately 8.5 kPa, nearly double that of Sn QSE (4.4 kPa), suggesting a notable improvement in mechanical robustness. This enhancement is attributed to a lower PDI index in Sn-FB QSE which forms a denser and more mechanically stable polymer network, implying its improvement in resisting sodium dendrite penetration.

### Experimental Characterizations and Theoretical Calculations of Ion Conductivity

**Fig. 2:**
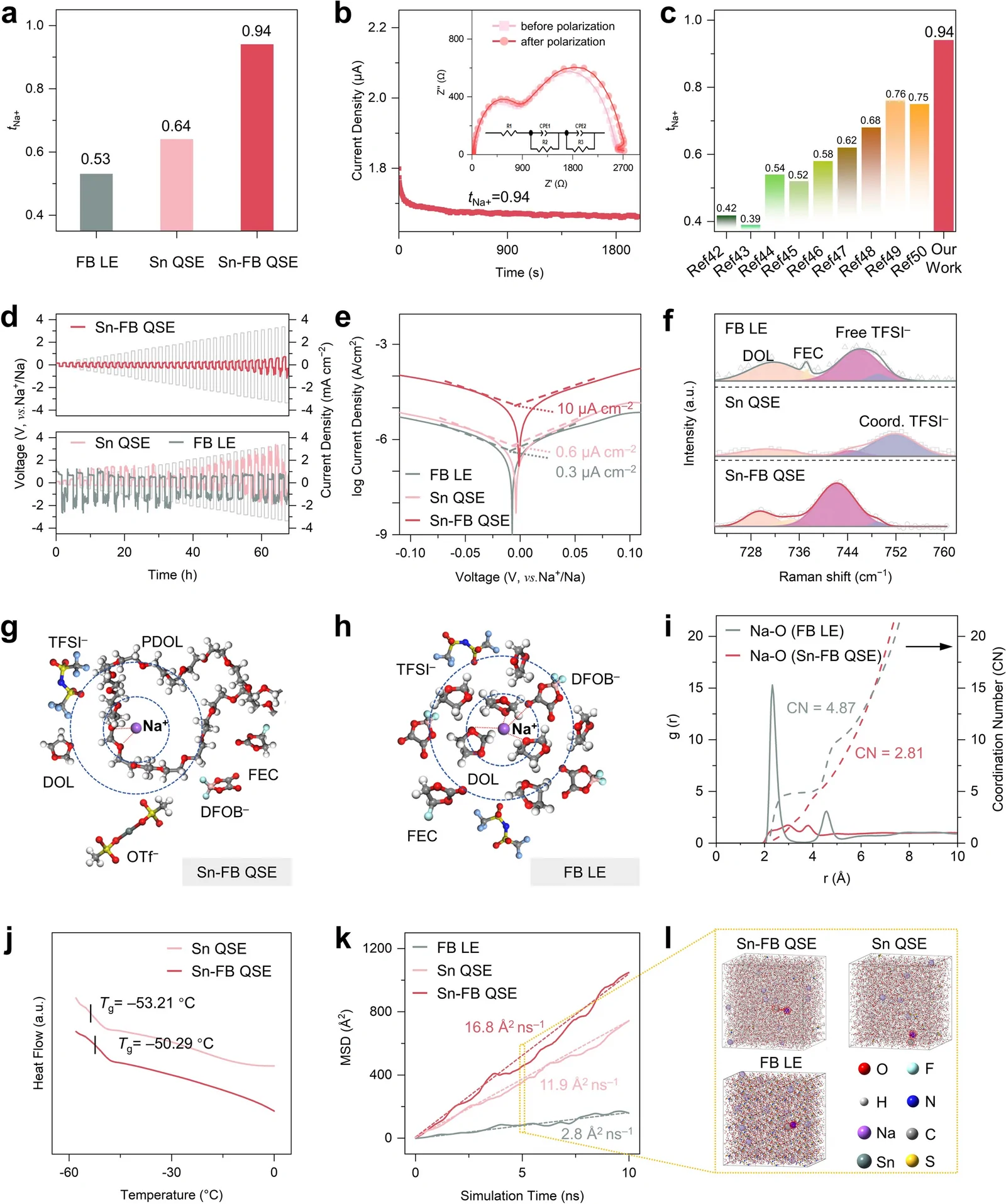
Electrochemical properties and theoretical calculations. **a** ${t}_{{\mathrm{Na}}^{+}}$ calculation of FB LE, Sn QSE, and Sn-FB QSE. **b** Direct current polarization curves of Sn-FB QSE (insert: corresponding Nyquist plots before and after polarization). **c** Comparison of ${t}_{{\mathrm{Na}}^{+}}$ between Sn-FB QSE and reported works [42–50]. **d** Critical current density, **e** Tafel curves, and **f** Raman spectra of FB LE, Sn QSE, and Sn-FB QSE. Snapshots from MD simulations of **g** Sn-FB QSE and **h** FB LE. **i** Calculated RDF g(r) and CN of Na–O in Sn-FB QSE and Sn QSE. **j** Glass transition behaviors (T_g_) of Sn QSE and Sn-FB QSE. Calculated MSD versus diffusion time for **k** Na^+^ diffusion and snapshots of FB LE, Sn QSE, and Sn-FB QSE at an interval of 5 ns

Ionic conductivity in the electrolyte arises from the transport of both Na^+^ and other anions. To better understand the Na^+^ transport behavior, ${t}_{{\mathrm{Na}}^{+}}$ is evaluated using the Bruce–Vincent method based on the current–time plot of Na||Na symmetric cells (Figs. 2a, b, and S4) [40, 41]. The ${t}_{{\mathrm{Na}}^{+}}$ values for FB LE, Sn QSE, and Sn-FB QSE were calculated to be 0.53, 0.64, and 0.94, respectively. Notably, the highest ${t}_{{\mathrm{Na}}^{+}}$ in Sn-FB QSE indicates single-conducting transport enabled by interlocked mediators, surpassing most reported works (Fig. 2c) [42–50]. In addition, Sn-FB QSE exhibits the highest *σ* of 1.3 mS cm^–1^, compared to 0.66 mS cm^–1^ in Sn QSE and 0.62 mS cm^–1^ in FB LE (Fig. S5). In Fig. 2d. Sn-FB QSE exhibits a low polarization voltage at low current density and achieves a high critical current density (CCD) of 3.0 mA cm^–2^, superior to both Sn QSE and FB LE, which contributes to the improved accommodation of high Na^+^ flux. This enhancement is further supported by Tafel analysis (Fig. 2e), where Sn-FB QSE displays an ultrahigh exchange current density of 10 μA cm^–2^, compared than that of 0.6 μA cm^–2^ in Sn QSE and 0.3 μA cm^–2^ in FB LE. These improvements imply a more stable interphase established in Sn-FB QSE. In addition, to assess their oxidative stability, the electrochemical stability windows (ESW) of all samples were measured (Fig. S6). Sn-FB QSE demonstrates the broadest ESW of 4.7 V (vs*.* Na^+^/Na), compared to the inferior ESW in Sn QSE (4.4 V) and FB LE (4.1 V), which can be attributed to the incorporation of interlocked mediators [32].

To better understand these electrochemical advantages, the coordination behavior of TFSI^–^ was examined via Raman spectra (Figs. 2f and S7). Free TFSI^–^ predominates particularly in FB LE and Sn-FB QSE, whereas coordinated (Coord.) TFSI^–^ is prevalent in Sn QSE, indicating an increase of free Na^+^ in the former [51]. This difference suggests enhanced Na^+^ dissociation due to the preferential coordination between DFOB^–^ and Na^+^ with anions anchored with strong Lewis-acid Sn^2+^ sites [32, 51]. Molecular dynamics (MD) simulations were further investigated to compare the Na^+^ coordination environment between Sn-FB QSE and FB LE by analyzing radial distribution functions (RDF) g(r) and coordination numbers (CN) of Na–O pairs. RDF results provide a molecular-level perspective on the Na^+^ solvation environment, where the changes in coordination peak intensities reveal how the mediators reorganize the solvation shell. As illustrated in Fig. 2g–i, the g(r) of Na–O (PDOL) pairs in Sn-FB QSE exhibits a weaker dominant peak at ~ 2.3 Å compared to Na–O (DOL), indicating that the Na–O (PDOL) bonds in Sn-FB QSE are weakened, which implies the introduction of PDOL reduces the overall solvation strength [32, 52]. Furthermore, the CN of Na–O (PDOL) pairs in Sn-FB QSE is 2.81, significantly lower than that in FB LE (4.87). This decrease in CN confirms the weaker Na^+^-O (PDOL) coordination in Sn-FB QSE, confirming more released free Na^+^ ions by incorporating PDOL. In addition, the introduction of DFOB^–^ and TFSI^–^ significantly reduces the binding energy of Na^+^-PDOL from − 3.216 to − 2.285 eV, facilitating rapid Na^+^ transport as calculated in Fig. S8.

The chain segment mobility between Sn QSE and Sn-FB QSE was compared by evaluating their glass transition temperatures (T_g_). Specifically, the T_g_ of Sn-FB QSE is at − 50.29 °C, approximately to that of − 53.21 °C in Sn QSE, indicating negligible differences in amorphous-state segmental mobility between the two systems (Fig. 2j) [53]. Here, the mean square displacement (MSD) calculations were applied to quantify the diffusion kinetics, with the significantly steeper slope for the Sn-FB QSE system directly confirming an accelerated Na^+^ diffusion coefficient (${D}_{{Na}^{+}}$) within the polymer matrix. In other words, Na^+^ diffusion is greatly facilitated in Sn-FB QSE as shown in Fig. 2k [54].Specifically, the ${D}_{{Na}^{+}}$ was found to be 16.8 Å^2^ ns^–1^ for Sn-FB QSE, higher than that of 2.8 Å^2^ ns^–1^ in FB LE, and 11.9 Å^2^ ns^–1^ in Sn QSE. This trend is visually corroborated by the Na^+^ displacement over an interval of 5 ns visualized in Fig. 2l, where Sn-FB QSE exhibits the most pronounced ion migration. These results clearly indicate that interlocking effect in bulk facilitates fastest Na^+^ diffusion in Sn-FB QSE, further supporting its ultrafast Na^+^ transport dynamics.

### Electrochemical Performances of Sn-FB QSE

**Fig. 3:**
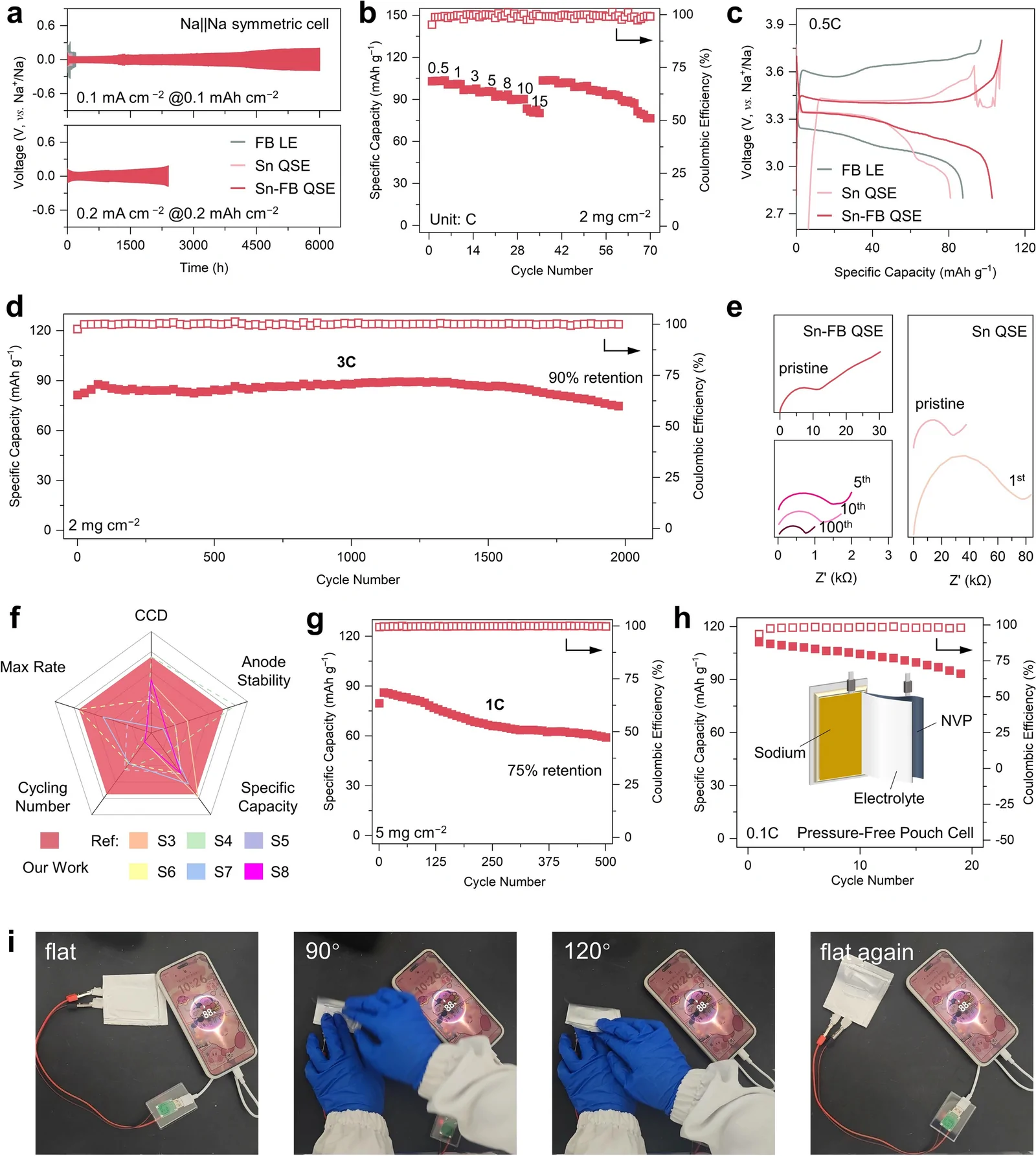
Electrochemical performances. **a** Galvanostatic cycling performance of FB LE, Sn QSE, and Sn-FB QSE symmetric cells at 0.1 and 0.2 mA cm^–2^, respectively. **b** Rate performance of Sn-FB GPE full cells ranged from 0.5 to 15C. **c** The first GCD curve at 0.5C of FB LE, Sn QSE, and Sn-FB QSE full cells. **d** Cycling performance of Sn-FB QSE full cells at 3C. **e** EIS curves comparison between Sn-FB QSE and Sn QSE full cells after different cycles at 0.5C. **f** Comparison between Sn-FB QSE cells and reported work. Cycling performance of Sn-FB QSE in **g** full cells (cathodic mass loading: 5 mg cm^–2^) at 1C and **h** pressure-free pouch cell at 0.1C. **i** Practical demonstration of pouch cell charging the phone during folding. Points plotted in **d** and **g** were taken every 25 cycles. The cells in **d** and **g** were all first activated at 0.5C for 5 cycles, which are not shown

The compatibility of various electrolytes with sodium metal was evaluated using Na||Na symmetric cells containing FB LE, Sn QSE, and Sn-FB QSE. To optimize the electrolyte, various amounts of Sn(OTf)_2_ initiator were tested (Fig. S9), confirming the optimal ratio for best performance. As shown in Fig. 3a, cells with Sn-FB QSE demonstrates remarkably stable Na^+^ plating/stripping performance, maintaining a low polarization of ~ 0.1 V for over 6000 h (~ 8.3 months). In contrast, cells with FB LE and Sn QSE displays large polarization and unstable cycling behavior, failing after only 50 h (0.1 mA cm^–2^ @ 0.1 mAh cm^–2^), which is attributed to poor Na^+^ transport [55]. Additionally, Sn-FB QSE exhibits excellent Na plating/stripping stability, sustaining over 2200 h (0.2 mA cm^–2^ @ 0.2 mAh cm^–2^). Even under higher current densities of 0.3, 0.5, and 1.0 mA cm^–2^, Sn-FB QSE still remains remarkable stability for 800, 400, and 210 h, respectively (Figs. S10–S12). Note that the voltage profiles temporarily fluctuated at around 1500 h (0.1 mA cm^–2^ @ 0.1 mAh cm^–2^) and 800 h (0.2 mA cm^–2^ @ 0.2 mAh cm^–2^) due to the power failure in our laboratory.

To investigate the practical applicability of Sn-FB QSE, full cells were assembled using NVP cathodes and sodium metal anodes. The amount of Sn-FB QSE was optimized between 20 and 80 μL at 0.1C (Fig. S13). An overdosed volume (70–80 μL) resulted in poor performance, while underfilling (20 μL) led to rapid capacity decline. A moderate 50 μL volume was found optimal and used in subsequent tests. Fig. 3b presents the rate capability performance of Sn-FB QSE under different current densities from 0.5 to 15C. It delivers an initial specific capacity as high as 103.4 mAh g^–1^ at 0.5C, which then remained high at 1, 3, 5, 8, 10, and 15C with specific capacities of 100.8, 97.5, 95.3, 89.5, 90.1, and 80.1 mAh g^–1^, respectively. Upon returning to lower current densities of 0.5C, the specific capacities recovered to 103.4 mAh g^–1^, confirming the excellent rate capability. In contrast, cells using FB LE and Sn QSE exhibit lower specific capacities and pronounced degradation as evidenced by the 1st galvanostatic charge/discharge (GCD) curves at 0.5C (Fig. 3c) [56]. Sn-FB QSE exhibits a significantly lower overpotential voltage compared to FB LE and Sn QSE. Moreover, Sn QSE fails to reach 3.8 V during the 1st charging process, which is attributed to the side reaction and electrolyte degradation.

The cycling capability and stability of Sn-FB QSE full cells are investigated as presented in Fig. S14a at the current density of 1C. After 2000 cycles, it maintains a high specific capacity of 80.2 mAh g^–1^ with 82% capacity retention. The corresponding GCD curves of Sn-FB QSE at 1C are plotted in Fig. S14b at selected intervals of 1^st^, 50^th^, 100^th^, 500^th^, and 1000^th^ cycle. It remains a low overpotential voltage of 0.16 V even after 1000 cycles compared to that of 0.19 V at the 1^st^ curve, which confirms the stable and fast Na^+^ transport in Sn-FB QSE full cells as well as their optimized interphases [57]. Even at a higher charging rate of 3C shown in Fig. 3d, the Sn-FB QSE full cell still maintains 90% capacity after 2000 cycles, retaining 73.1 mAh g^–1^. When the current density soars to 5C, the Sn-FB QSE full cell keeps retaining a high specific capacity of 53.4 mAh g^–1^ after 800 cycles (Fig. S15), indicating the rapid Na^+^ transport and ultra-stable interphase in the Sn-FB QSE full cell. Meanwhile, the electrochemical impedance spectroscopy (EIS) is then implemented to probe the superior Na^+^ transport and optimized interphases in Sn-FB QSE compared to Sn QSE in full cells (Fig. 3e). Both Sn-FB QSE and Sn QSE exhibit a large initial impedance in the pristine state. However, Sn-FB QSE displays a decreasing impedance over cycling (5^th^, 10^th^, and 100^th^ cycles), indicating the formation of a stable electrode–electrolyte interphase and facilitating rapid Na^+^ transport. Contrarily, Sn QSE delivers a sharp increase in impedance after the 1st charge due to severe electrolyte decomposition [56].

To verify the superiority of interlocked mediator engineering, we compare several key metrics (CCD, anode stability, specific capacity, cycling number, and max rate) of our Sn-FB QSE with other reported works in Fig. 3f (details in Table S3), in which our Sn-FB QSE exhibits a remarkable and excellent performance in all aspects. These findings underscore the superior enhancement of achieving a single-ionic-conducting QSE with ultra-stable interphases by dual interlocked mediator engineering.

More extensively, high-mass-loading NVP (5 mg cm^–2^) full cells are demonstrated in Fig. 3g and achieve stable cycling performance, maintaining 75% capacity retention after 500 cycles at 1C. Meanwhile, a pressure-free pouch cell (4 × 5 cm^2^) was assembled with Sn-FB QSE (Fig. 3h) and retained a high specific capacity of 93.3 mAh g^–1^ (84% capacity retention) after 19 cycles at 0.1C with an average Coulombic efficiency of 98% from the 2^nd^ cycle, emphasizing its superiority in ultra-stable interphases. To further verify its practicality, we used one pouch cell to charge the phone as presented in Fig. 3i. Despite repeated full folding, it can continuously power the phone (details in Video S1), which demonstrates its superior flexibility. In addition, the Sn-FB QSE pouch cell retains 60% capacity after 100 cycles under a minimal clamping force of 100 kPa at 0.2C (Fig. S16).

Beyond NVP, we also extend the cathode selection to NaNi_1/3_Fe_1/3_Mn_1/3_O_2_ (NFM) with a high mass loading of 17.54 mg cm^–2^ within the voltage range of 2.0–4.0 V. In Fig. S17, this NFM-based full cell delivers 129.9 mAh g^–1^ at 0.1C during the initial 3 cycles and retains a high specific capacity of 108.9 mAh g^–1^ at 0.2C after a total of 17 cycles.

### Highly Adaptable Bilateral Interphases

**Fig. 4:**
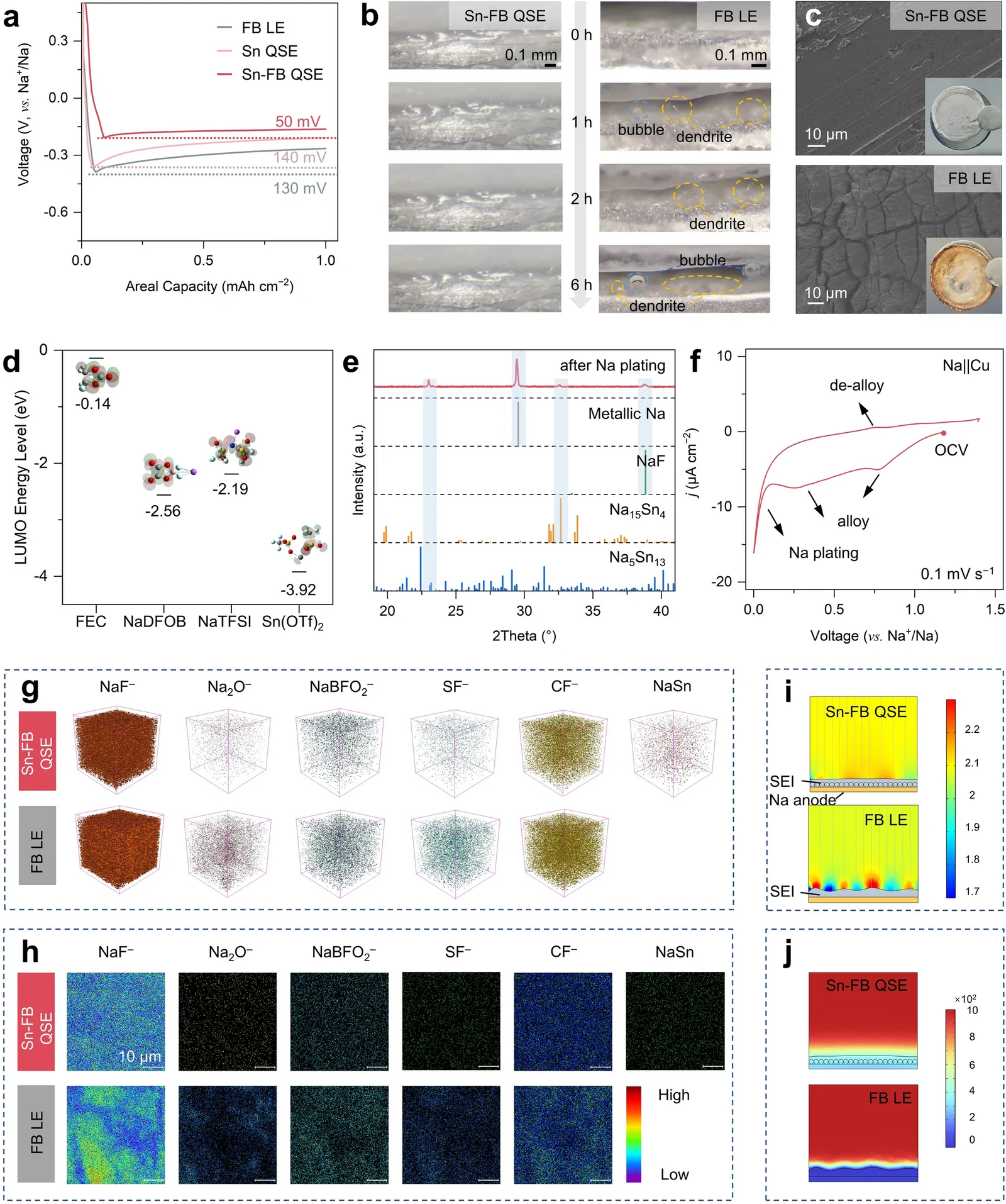
Sodium anode–electrolyte interface stability. **a** Nucleation overpotentials of sodium with FB LE, Sn QSE, and Sn-FB QSE. **b** Cross-sectional optical images of the sodium anode with Sn-FB QSE and FB LE at 0.1 mA cm^–2^. **c** SEM images of the sodium anode with Sn-FB QSE and FB LE after deposition (insertion: corresponding optical photos). **d** LUMO energy levels of FEC, NaDFOB, NaTFSI, and Sn(OTf)_2_ interacting with Na^+^. **e** XRD patterns of the sodium anode after deposition. **f** The 1st CV curve of Na||Sn-FB QSE||Cu cell at 0.1 mV s^–1^. ToF–SIMS **g** 3D and **h** 2D rendering visualizations of the selected fragments of Sn-FB QSE (upper) and Sn QSE (lower). Finite element simulation of **i** electric filed and **j** Na^+^ flux distributions of Sn-FB QSE (upper) and Sn QSE (lower) on SEI

Dual interlocked mediator engineering in Sn-FB QSE not only enhances electrochemical performance but also delivers highly adaptable bilateral interphases. It promotes sodium deposition at the 1^st^ cycle, which then facilitates better SEI formation. To evaluate this, the nucleation overpotentials of various electrolytes were measured using Na||Cu half-cells (Fig. 4a). Sn-FB QSE demonstrates the lowest nucleation overpotential of only 50 mV, highlighting its superior ability to facilitate Na^+^ nucleation. In contrast, FB LE and Sn QSE exhibit high nucleation overpotentials of 130 and 140 mV, respectively, indicative of difficult Na^+^ nucleation. Furthermore, in situ optical microscopy was employed to directly observe sodium deposition behavior in Sn-FB QSE and FB LE as presented in Fig. 4b (current density = 0.1 mA cm^–2^). In Sn-FB QSE, no dendrite formation or bubble was observed during a continuous 6 h plating period, indicating abundant sodiophilic sites as well as enhanced stable and uniform Na^+^ deposition. Conversely, in FB LE, dendrite growth and bubble formation were evident after only 1 h, and these features worsened significantly between 2 and 6 h, suggesting poor SEI stability and nonuniform ion transport. Morphological differences were further compared operating scanning electron microscopy (SEM) (Fig. 4c). After the 5^th^ sodium deposition, the sodium metal surface remained smooth and compact, with minimal roughening, as shown in the inserted optical image. Elemental mapping confirms the uniform presence of Sn on the deposited surface, suggesting effective interfacial incorporation of the Sn species (Fig. S18). In contrast, the Na surface in FB LE appears rough, cracked, and turns brown (inserted optical image), indicative of uneven deposition and unstable interphase formation [4].

To assess the reduction tendency of individual electrolyte components, the LUMO energy levels of FEC, NaDFOB, NaTFSI, and Sn(OTf)_2_ are compared in Fig. 4d. Among these, Sn(OTf)_2_ exhibits the lowest LUMO energy level at − 4.87 eV, followed by NaDFOB at − 2.56 eV. This difference in reduction tendency suggests that Sn^2+^ is the most readily reduced species, which is likely to undergo initial reduction at the sodium metal surface, potentially forming NaSn alloys and serving as sodiophilic sites [25, 58]. To validate this hypothesis, the composition of the sodium anode after 6 h of Na^+^ plating in Sn-FB QSE was analyzed by X-ray diffraction (XRD) (Fig. 4e). In addition to the characteristic peak at 29.4° corresponding to the metallic sodium (PDF #00-001-0850), characteristic peaks at 23.0° and 32.7° are assigned to Na_5_Sn_13_ (PDF #04-007-2431) and Na_15_Sn_4_ (PDF #030-065-9654), respectively, confirming the formation of NaSn alloy phases on the sodium surface. Notably, the characteristic peak at 38.8° is attributed to NaF (PDF #00-036-1455), which is known as a key SEI component due to its robustness and fast ion transport properties [59]. The NaSn alloy formation mechanism was further elucidated by conducting the 1^st^ cyclic voltammetry (CV) in the Na||Cu half-cell at a scanning rate of 0.1 mV s^–1^ (Fig. 4f). During the 1^st^ cathodic scanning, the reduction peaks at 0.8 and 0.3 V (vs*.* Na^+^/Na) are observed, corresponding to the stepwise NaSn alloying reactions between Na and Sn species. A subsequent oxidation peak at 0.8 V is denoted as the de-alloy process. Additionally, a sharp peak at 0.1 V indicates the metallic Na plating progress.

The SEI component and its distribution were further analyzed using time of flight secondary ion mass spectrometry (ToF–SIMS) in Fig. 4g, h. The ToF–SIMS 3D rendering visualizations of Sn-FB QSE and FB LE (Fig. 4g) reveal a high concentration of the inorganic component of NaF^–^ in both samples. Notably, in Sn-FB QSE, signals corresponding to NaSn alloy species are also detected within the SEI layer. This result indicates the formation of a hybrid NaSn alloy/inorganic-rich SEI, which is beneficial for lowering nucleation overpotentials and boosting Na^+^ transport. In addition, Sn-FB QSE exhibits suppressed formation of organic degradation products such as SF^–^ and CF^–^, indicating the reduced concentration polarization, while diminished presence of Na_2_O^–^, NaBFO_2_^–^ species implies improved anion retentions at the anode and restrained side reactions [60, 61]. In Figs. 4h and S19, ToF–SIMS 2D rendering visualizations confirm a more uniform distribution of each SEI component in Sn-FB QSE compared to FB LE. This homogeneity indicates a more evenly distributed SEI morphology, intentionally facilitating stable Na plating/stripping behavior, which is attributed to the homogenized electric field.

Finite element simulations were conducted to evaluate the electric field and Na^+^ flux distribution across the SEI layers in Sn-FB QSE and FB LE. Note that the ball-like particle represents NaSn alloy. As illustrated in Fig. 4i, the SEI formed in Sn-FB QSE exhibits a significantly more uniform electric field distribution compared to that of FB LE. This improvement is attributed to the introduction of a hybrid NaSn alloy/inorganic-rich SEI, which enhances interfacial conductivity and electric field homogeneity. The corresponding Na^+^ flux distribution (Fig. 4j) reveals a more uniform and intensified ion transport across the SEI in Sn-FB QSE, in contrast to the uneven and lower Na^+^ flux observed in FB LE. Overall, these results suggest that the interlocked mediator engineering promotes the formation of a hybrid NaSn alloy/inorganic-rich SEI at the anode, homogenizing even and fast electric fields as well as inducing uniform, fast Na^+^ transport during cycling.

**Fig. 5:**
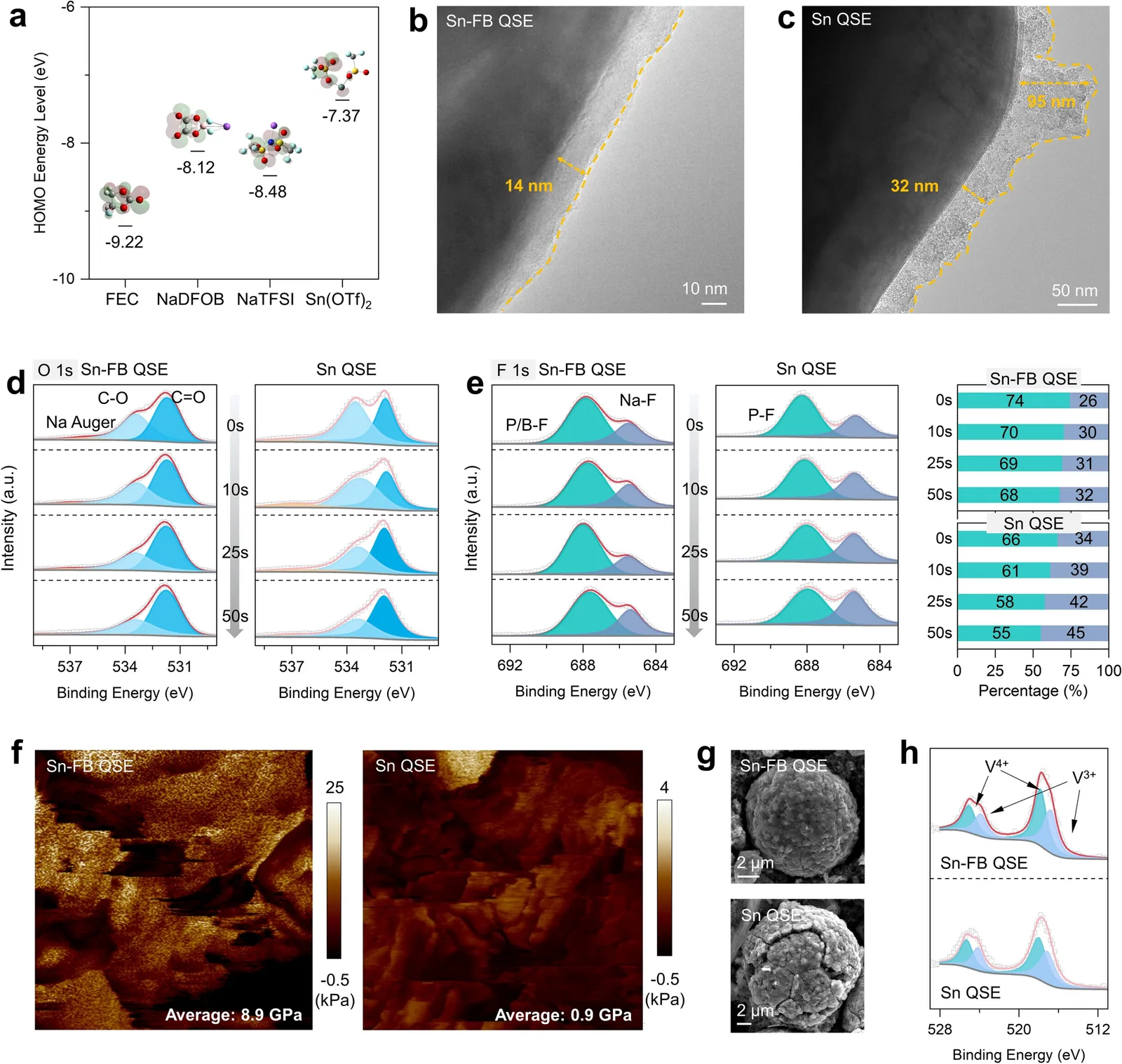
Cathode–electrolyte interface stability. **a** HOMO energy levels of FEC, NaDFOB, NaTFSI, and Sn(OTf)_2_ interacting with Na^+^. TEM images of CEI on the cycled NVP cathode of **b** Sn-FB QSE and **c** Sn QSE. XPS depth profiles of **d** O 1*s* and **e** F 1*s* as well as the corresponding ratio of the CEI formed on the NVP cathode of Sn QSE and Sn-FB QSE. **f** AFM images of Young’s modulus and **g** SEM images of cycled NVP cathode for Sn-FB QSE and Sn QSE. **h** V 2*p* XPS profiles of cycled NVP cathode for Sn-FB QSE and Sn QSE. (cycling condition: 0.5C for 300 cycles)

To assess the oxidation propensity of individual electrolyte components at the cathode, the highest occupied molecular orbital (HOMO) energy levels of FEC, NaDFOB, NaTFSI, and Sn(OTf)_2_ were compared (Fig. 5a). Among these, NaDFOB exhibits a high HOMO energy level at − 8.12 eV, following Sn(OTf)_2_ (− 7.37 eV), which indicates that NaDFOB is susceptible to oxidation and is likely to undergo initial oxidative decomposition at the cathode. The morphology and thickness of the CEI layers in both Sn QSE and Sn-FB QSE were investigated using transmission electron microscopy (TEM). As shown in Fig. 5b, the CEI formed in Sn-FB QSE is notably thinner and more uniform, with a consistent thickness of only 14 nm. Furthermore, well-preserved lattice fringes observed in Sn-FB QSE (Fig. S20) confirm minimal structural degradation of the cathode, suggesting improved interface stability and reduced side reaction [31, 62]. In contrast, the CEI layer formed in Sn QSE is obviously uneven, with a thickness ranging from 32 to 95 nm (Fig. 5c).

The compositions of CEI layers in both Sn-FB QSE and Sn QSE were further elucidated by applying in-depth X-ray photoelectron spectroscopy (XPS) at the interval of 0, 10, 25, and 50 s (etching rate = 0.2 nm s^–1^). As shown in Fig. 5d, the peaks at 532 eV correspond to the C=O bonds, while the peak at 534 eV corresponds to C–O bonds derived from the polymer matrix. With increasing etching time, the intensity of the C–O component gradually decreases in both Sn-FB QSE and Sn QSE, indicating the reduction of organic species in the inner CEI layers. Notably, the C–O component ratio is consistently less intense in Sn-FB QSE, which corresponds to that of C 1*s* (Fig. S21). This trend indicates the suppressed PDOL matrix degradation in Sn-FB QSE. Note that the broaden peak at 537 eV is denoted as the Na Auger peak. In the F 1*s* spectra (Fig. 5e), the peak at 688 eV is assigned to P–F in Sn QSE, whereas in Sn-FB QSE, the same region also includes contributions from B–F bonds originating from the decomposition of NaDFOB [63]. The peak at 685 eV is denoted as Na–F in both Sn-FB QSE and Sn QSE, indicating the presence of this single-ion-conductive inorganic CEI component. To quantify the evolution of fluorinated species, the relative content of F 1s components was tracked over etching time. The ratio of P–F/B–F species in Sn-FB QSE ranges of 74%–68% as etching goes, while it decreases rapidly to 66%–55% in Sn QSE. This obviously higher ratio of P–F/B–F component in Sn-FB QSE confirms the successful formation of a more stable, higher ion-conductive and inorganic-rich CEI.

In Fig. 5f, atomic force microscopy (AFM) was applied to compare the Young’s modulus of CEI layers formed in Sn-FB QSE and Sn QSE. The CEI formed in Sn-FB QSE exhibits an average Young’s modulus of 8.9 GPa, 10 times higher than that in Sn QSE (0.9 GPa), indicating the significantly enhanced mechanical strength. Further insight into structural integrity was obtained from postmortem SEM images of NVP particles (Figs. 5g and S22). In Sn-FB QSE, the particles largely retain their pristine morphology with minimal cracking, attributing to the robust CEI, while those in Sn QSE endure severe cracking after cycling. Accordingly, the valence states of Vanadium were analyzed by conducting V 2*p* XPS (Fig. 5h) [64, 65]. The peaks at 516 and 524 eV represent V^3+^, whereas the peaks at 518 and 528 eV represent V^4+^. A higher proportion of V^3+^ was oxidized to V^4+^ in Sn-FB QSE, indicating more complete and efficient reactions and enhancing Na^+^ storage performance. Note that an enhanced QSE–electrode contact is attributed to the retarder. Therefore, a thin yet robust CEI is formed due to the dual interlocked mediator engineering, promoting the preserved structural integrity and enhanced Na^+^ transport kinetics during cycles.

## Conclusions

In this work, a single-ion-conducting QSE with highly adaptable interphases was developed via a dual interlocked mediator engineering, incorporating Sn^2+^ salt as a in situ cationic polymerization initiator and DFOB^–^ as a retarding agent to suppress the runaway polymerization in a PDOL-based system. This interlocking effect in Sn-FB QSE bulk not only yields a homogenous and mechanically robust network but also achieves ${t}_{{\mathrm{Na}}^{+}}$ = 0.94 by dissociating Na^+^-TFSI^–^ and reducing Na–O interactions. Consequently, the other interlocking effect enables adaptable bilateral interphases. During cell operation, a hybrid NaSn alloy/inorganic-rich SEI was induced by Sn^2+^ together with other electrolyte species, which homogenizes the electric field and facilitates rapid Na^+^ transport and enables no-dendrite Na^+^ plating/stripping with low polarization for over 6000 h. Meanwhile, DFOB^–^ oxidizes at the cathode to generate a thin yet robust CEI, mitigating electrolyte degradation and lowering interfacial Na^+^ diffusion resistance. As a result, the NVP||Sn-FB QSE||Na full cells deliver 80.1 mAh g^–1^ at an ultrafast rate at 15C and achieve 90% capacity retention after 2000 cycles at 3C. Furthermore, a pressure-free pouch cell successfully operates for 19 cycles, and Sn-FB QSE is also compatible with high-mass-loading cathode NVP (5 mg cm^–2^) and NFM (17 mg cm^–2^), which underscores the potential of dual interlocked mediator engineering for practical ultrafast-charging and long-life SMBs.

## Supplementary Information

Below is the link to the electronic supplementary material.

Supplementary file1 (DOCX 3758 kb)

Supplementary file2 (MP4 629 kb)
