# Supplementary material for: Dual Interlocked Mediators Enable Single-Ion-Conducting Quasi-Solid-State Electrolytes for Ultrafast-Charging Long-Life Sodium Metal Batteries
Source: Nanomicro Lett. 2026 May 21;18:381. doi: 10.1007/s40820-026-02236-2 (PMC13190910; doi:10.1007/s40820-026-02236-2)
Supplement: Supplementary file 1 — Supplementary file1 (DOCX 3758 kb) [file 40820_2026_2236_MOESM1_ESM.docx]

Supporting Information for

**Dual Interlocked Mediators Enable Single-Ion-Conducting Quasi-Solid-State Electrolytes for Ultrafast-Charging Long-Life Sodium Metal Batteries**

Yuan Zhang^1,^ Long Pan^1,^*, CHEONG WA LEONG^1^, Xing-Guo Qi^2^, Xiaozhong Huang^1^, Xinyi Cai^1^, Mufan Cao^1^, Min Gao^1^, Haoyu Zhang^1^, Dawei Sha^3^, Yang Zhou^1,^*, ZhengMing Sun^1,^*

^1^ State Key Laboratory of Engineering Materials for Major Infrastructure, School of Materials Science and Engineering, Southeast University, Nanjing 211189, P. R. China

^2^ HiNa Battery Technology Co., Ltd., Beijing 213300, P. R. China

^3^ Institute of Technology for Carbon Neutralization, College of Electrical, Energy and Power Engineering, Yangzhou University, Yangzhou 225009, P. R. China

* Corresponding authors. E-mail: [panlong@seu.edu.cn](mailto:panlong@seu.edu.cn) (Long Pan); [tomaszy@seu.edu.cn](mailto:tomaszy@seu.edu.cn) (Yang Zhou); [zmsun@seu.edu.cn](mailto:zmsun@seu.edu.cn) (ZhengMing Sun)

#### S1 Experimental Section

***Electrochemical Measurements.*** CR2032 coin-type cells were assembled in an argon-filled glove box for both symmetric cells and full cells. To assemble Na symmetric cells, Na metal foils (0.45 mm, Changjing Co. Ltd) were used as the working electrodes and Al_2_O_3_@PE (16 μm) was attached as the separator with electrolyte injected. In full cells, Na_3_V_2_(PO_4_)_3_ (NVP, Canrd Co.Ltd) and NaNi_1/3_Fe_1/3_Mn_1/3_O_2_ (17.45 mg cm^–2^, NFM, HiNa Battery Co. Ltd) were employed as cathode electrodes with electrolyte injected. To prepare NVP electrodes, NVP, conductive additive (carbon black), and binder (polyvinylidene fluoride) were mixed in *N*-methylpyrrolidone with the mass ratio of 8: 1: 1 to form a homogenous slurry. The slurry was coated on carbon coated Al foil and then vacuum-dried at 80°C for 12 h. The average mass loadings of active materials are approximately 2~3 mg cm^−2^ for NVP electrodes (diameter = 10 mm). The specific capacity of full cells was calculated based on the mass of cathode. Galvanostatic charge-discharge (GCD) tests were performed on a LAND-CT2001C tester (Wuhan Lanhe). Full cells were tested in the voltage range of 2.8−3.8 V. All cells were stood for at least 48 h before testing to ensure fully polymerization. All electrochemical tests were carried out at 25 °C.

***Materials Characterization.*** Fourier transform infrared (FT-IR) spectra were acquired by using a Thermo Scientific spectrometer. Pressurized water ^1^H nuclear magnetic resonance (NMR) spectra were obtained by using Agilent 800 MHz with DMSO-d6. Gel permeation chromatography (GPC) was acquired by Agilent GPC 50. Mechanical properties were tested by using MST E43.104. Raman spectra were collected using a WITec Alpha 300R spectrometer with a laser wavelength of 532 nm at a power of 0.14 mW. In-situ electrochemical optical microscope was applied by using Biologic SP-150 coupled to Zeiss optical microscope. X-ray photoelectron spectroscopy (XPS) measurements were performed using a Thermo Scientific K-Alpha spectrometer with Al Kα radiation. In-depth XPS sputtering rate is 2 nm s^–1^. Scanning electron microscopy (SEM) images were acquired using an argon-protected JEOL JSM-IT800 with an accelerating voltage of 5.0−15.0 kV. X-ray diffraction (XRD) patterns were recorded using a Haoyuan DX-2700BH diffractometer. Time of flight secondary ion mass spectrometry (ToF-SIMS) was applied using PHI nano TOF3 instrument with 0.75 nm/s (sputtering rate). Transmission electron microscopy (TEM) and high-resolution TEM (HRTEM) images were obtained using a Thermo Fisher Talos F200X microscope with an accelerating voltage of 200 kV. Atomic forced microscopy (AFM) was operated by using Dimension ICON.

***Computational Methods.*** The finite element analysis simulations are conducted with the COMSOL Multiphysics software. The FEA model was defined by the law of mass conservation and electroneutrality assumption of related ions without considering the possible side reactions. Simulation considers the influence of two different factors on Na^+^ concentration: concentration diffusion and electric field migration. A simulation model was built for a local model of 8*9um, with an initial layer of 0.6um. The simulation considers the effects of two different electrolytes. The first is FB-LE. The second is Sn-FB QSE, which has a layer of Na_15_Sn_4_ alloy on the surface, allowing for regulation of the surface electric field and inducing uniform and rapid Na^+^ flux. Using the current distribution and concentration diffusion equation to track the current and concentration distribution, the Na^+^ flux Ni follows the Nernst-Planck expression:


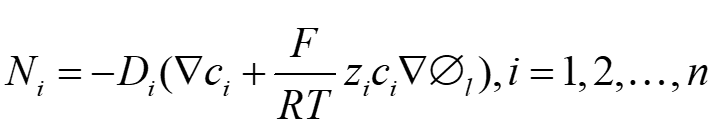


where *D_i_*, *N_i_*, c*_i_*, z*_i_*, *F*, *T*, *R*, and ϕ*_l_* denote the diffusion coefficient, the flux, the concentration, the charge number, the Faradaic constant, temperature, gas constant, and electrolyte potential, respectively. *l* stands for the position along the diffusion region of thickness d (0 < l < d). Based on the steady-state continuity equations and the law of mass conservation, we have


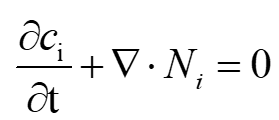


and the electroneutrality assumption was represented as


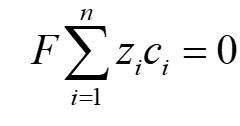


The boundary condition was set as followed:


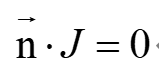


The simulation steps include a current distribution initialization and a steady step. The initial ion concentration is set to 1000 mol m^–3^. And the boundary potential is 0 V. During the deposition process, ions arriving at the growing surface are reduced to Na atoms, thickening the deposit and depleting nearby electrolyte. Thus, the local surface concentration tends toward 0, while ion flux and electron density peak at dendrite tips.

DFT calculation was carried out using Gaussian 16 software. Geometrical optimizations and frequency analyses employed B3LYP method and 6-311++g(d,p). The HOMO-LUMO orbitals were obtained and visualized in GaussView 6.0 software. The calculations of binding energy and electrostatic potential were operated follow the process: mapping of molecular structures—optimization of conformational and frequency—calculations—data organization and analysis. Based on density-functional theory DFT calculations, GaussView 6.0 and Gaussian 16 were applied to optimize the conformation and calculate the frequency of the compounds [S1]. This conformational optimization and frequency calculation were performed at the b3lyp/6-31g level of theory.

Three distinct simulation systems were constructed, with the coordination environments detailed in Table S1. Energy minimization was first performed using the conjugate gradient algorithm, with a maximum of 10,000 iterations and convergence criteria set to 1.0 × 10^-6^ for both energy and force. The systems were then equilibrated under the NPT ensemble at 300 K and 1 atm for 500 ps to achieve volume and pressure stability. Following equilibration, long-timescale simulations were carried out in the NVT ensemble at 300 K for 10 ns to investigate the diffusion behavior of Na⁺. All intermolecular interactions were described using the OPLS_AA force field, and all molecular dynamics simulations were performed using the LAMMPS software package.

**Table S1** Initial configuration of the systems

|  | NaTFSI | NaDFOB | FEC | Sn(OTf)_2_ | PDOL | DOL |
| --- | --- | --- | --- | --- | --- | --- |
| Sys1  (Sn-FB QSE) | 10 | 4 | 1 | 1 | 60 | 10 |
| Sys2  (FB LE) | 10 | 2 | 1 | 0 | 0 | 67 |
| Sys3  (Sn QSE) | 10 | 0 | 1 | 1 | 37 | 30 |

#### S2 Supplementary Figures and Tables


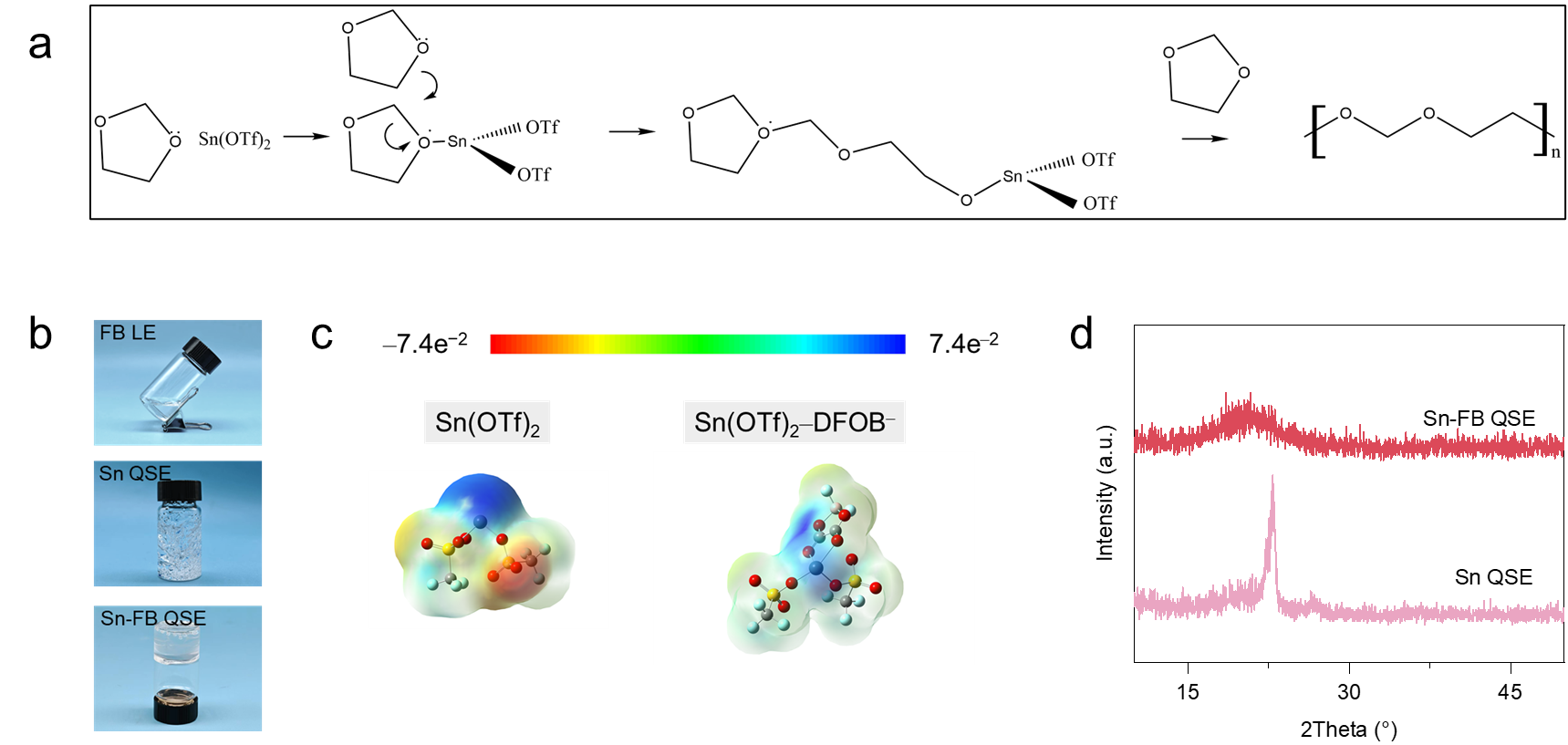


**Fig. S1 a** Open-loop polymerization process illustration [S2], **b** digital photos of FB LE, Sn QSE and Sn-FB QSE. **c** electrostatic potential distribution based on DFT calcuation of Sn(OTf)_2_ and Sn(OTf)_2_-DFOB⁻, and **d** XRD patterns of Sn-FB QSE and Sn QSE.


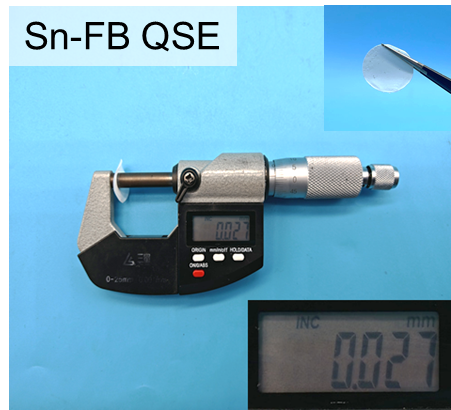


**Fig. S2** Thickness calibration of Sn-FB QSE electrolyte using a vernier caliper (insert: Sn-FB QSE electrolyte).


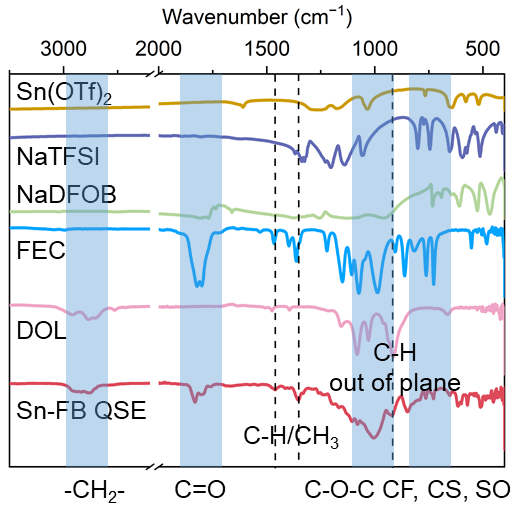


**Fig. S3** FT-IR spectra of Sn(OTf)_2_, NaTFSI, NaDFOB, FEC, DOL, and Sn-FB QSE.

**Table S2** M_w_ and M_n_ of Sn-FB QSE and Sn QSE

| **Sample** | **M_w_** | **M_n_** |
| --- | --- | --- |
| Sn QSE | 562681 | 123699 |
| Sn-FB QSE | 48783 | 30634 |


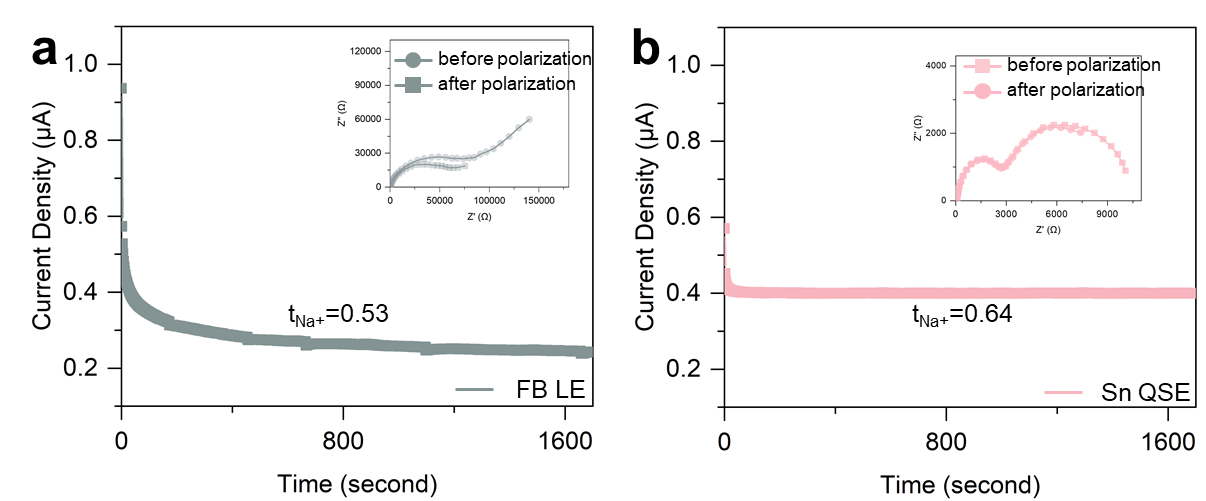


**Fig. S4** $\text{t}_{\text{Na}^{\text{+}}}$ calculation of (a) FB LE and (b) Sn QSE.


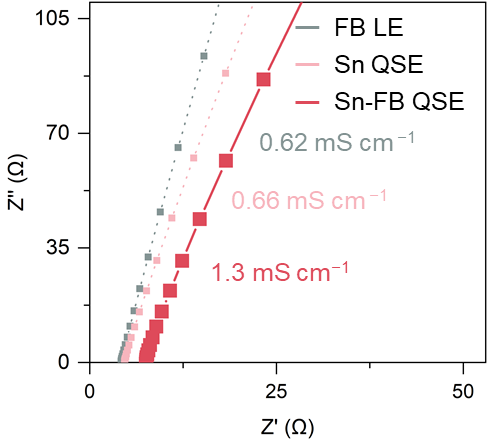


**Fig. S5** *σ* of FB LE, Sn QSE and Sn-FB QSE.


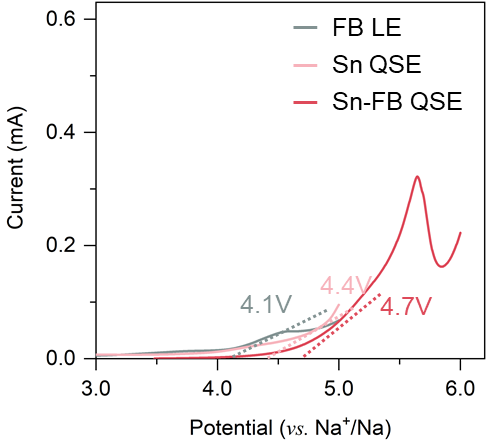


**Fig. S6** Electrochemical stability window (V) of FB LE, Sn QSE and Sn-FB QSE (scanning rate= 1 mV s⁻^1^ from 3V *vs.* Na^+^/Na).


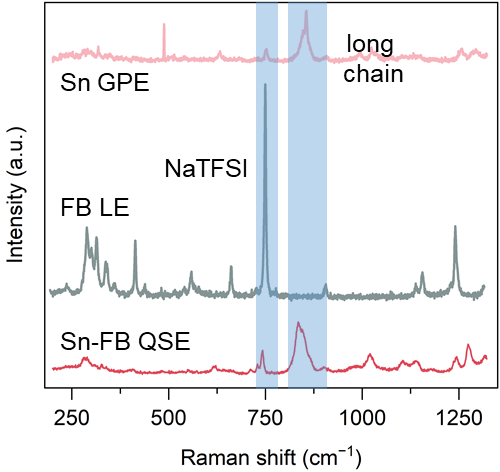


**Fig. S7** Raman spectra of Sn QSE, FB LE, and Sn-FB QSE.


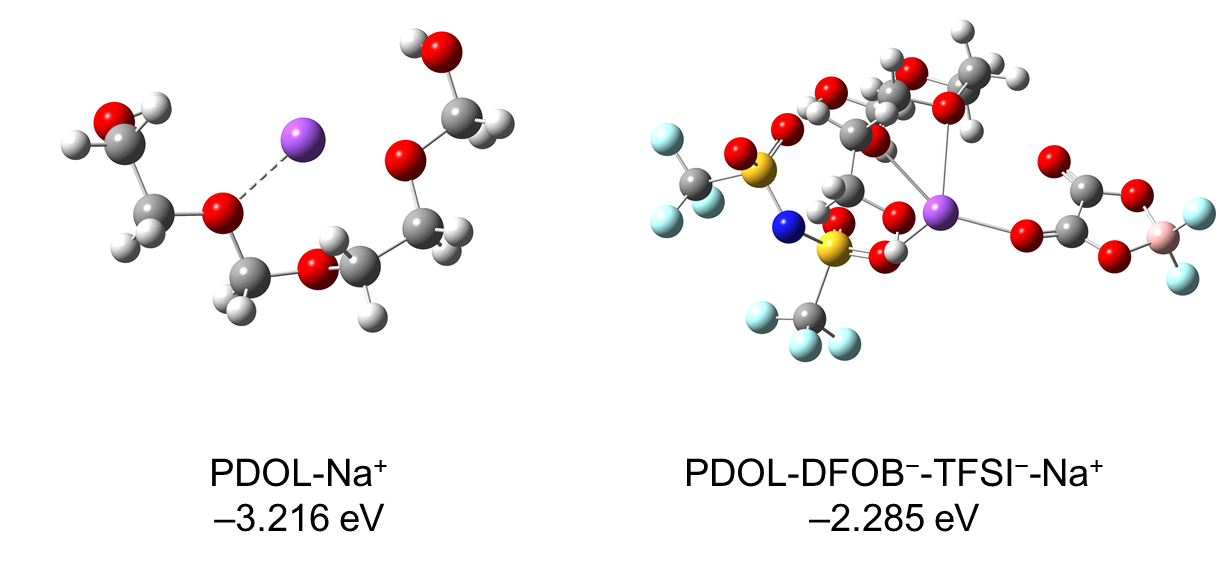


**Fig. S8** Calculated binding energies of PDOL-Na^+^ before and after introducing DFOB⁻ and TFSI⁻.


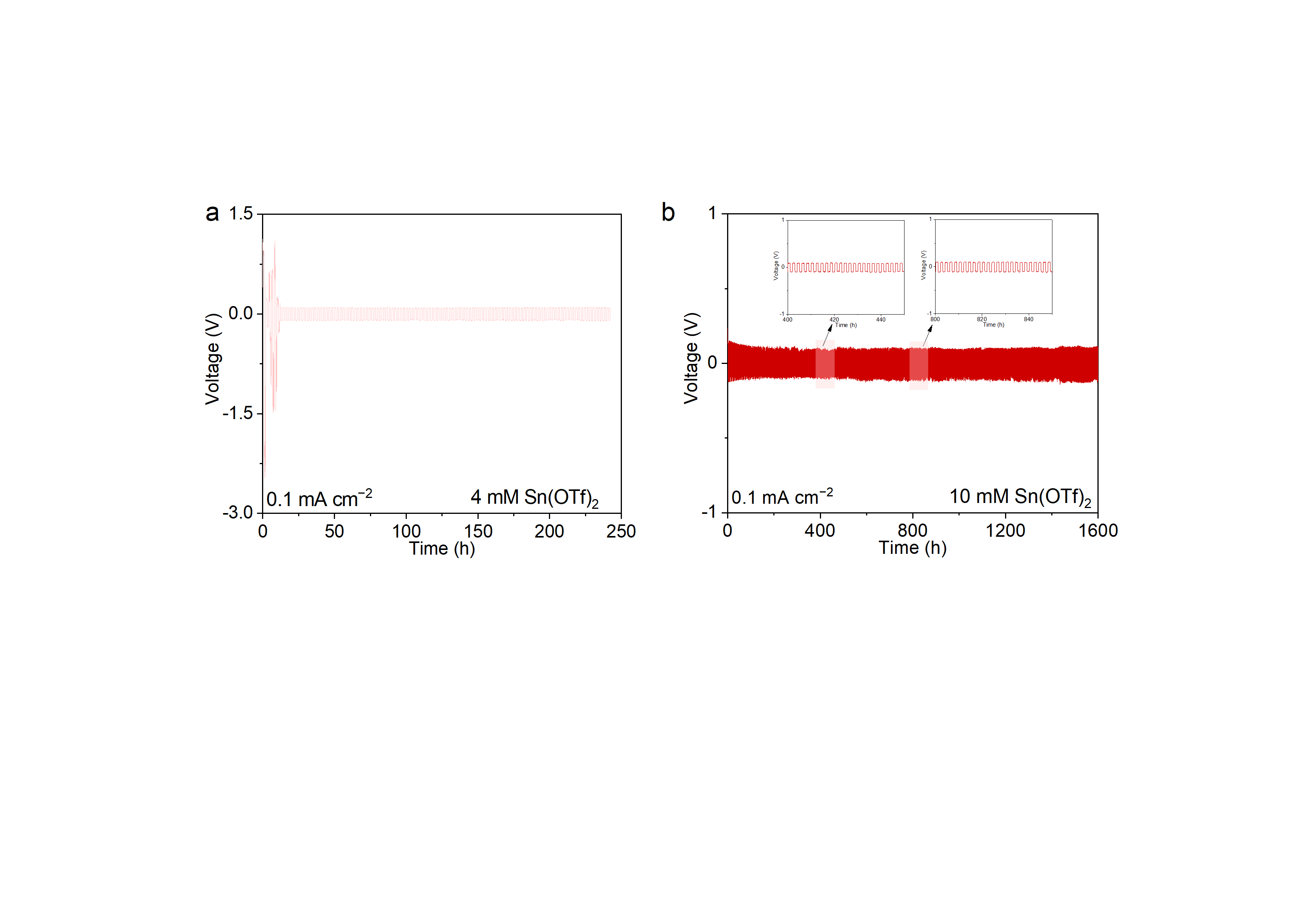


**Fig. S9** Cycling performance of varied amount initiator in Na symmetric cell at 0.1 mA cm^−2^, 0.1 mAh cm^−2^. a) 4 mM Sn(OTf)_2_; b) 10 mM Sn(OTf)_2_

To optimize the electrolyte, different amounts of Sn(OTf)_2_ were tested. When 4 mM Sn(OTf)_2_ was added to synthesize Sn-FB QSE, it went through short circuit quickly and fail to avoid sodium dendrite due to the unstable interphase and the inferior robustness. However, too much Sn(OTf)_2_ (10 mM) introduced also led to the unstable Na^+^ plating/stripping. In our case, the optimal ratio of Sn(OTf)_2_ is 6 mM.


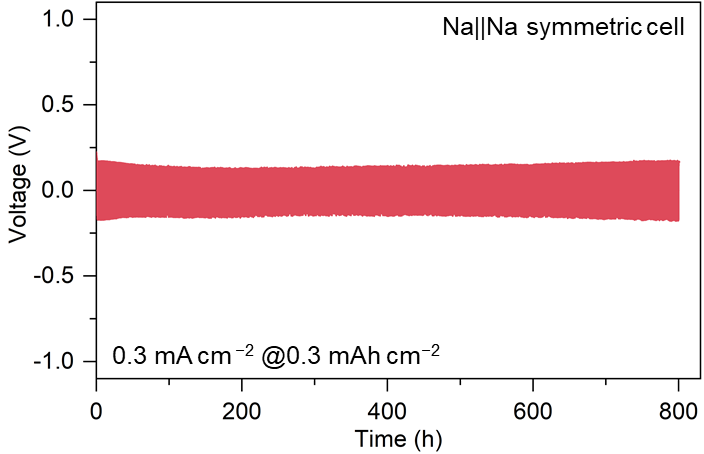


**Fig. S10** Cycling performance of Sn-FB QSE in Na||Na symmetric cell at 0.3 mA cm^−2^ @ 0.3 mAh cm^−2^.


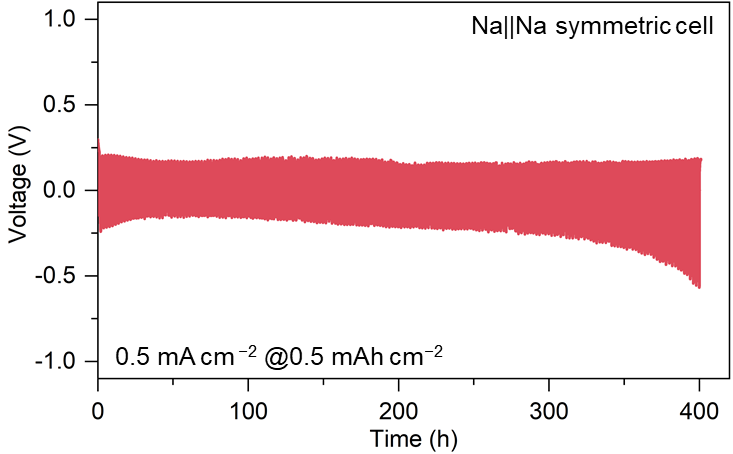


**Fig. S11** Cycling performance of Sn-FB QSE in Na||Na symmetric cell at 0.5 mA cm^−2^ @ 0.5 mAh cm^−2^.


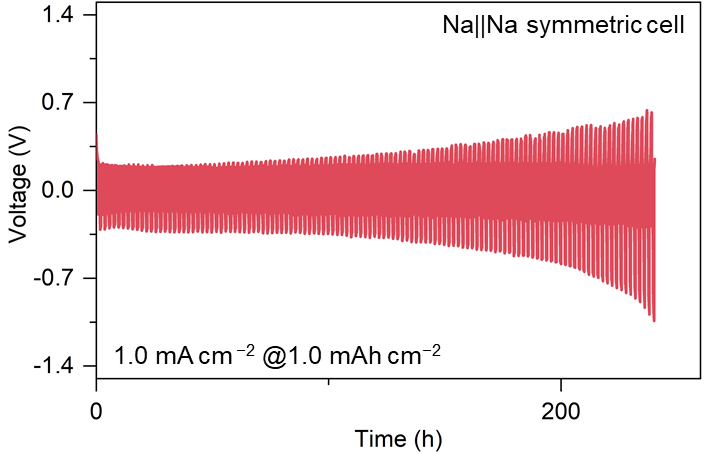


**Fig. S12** Cycling performance of Sn-FB QSE in Na||Na symmetric cell at 1.0 mA cm^−2^ @ 1.0 mAh cm^−2^.


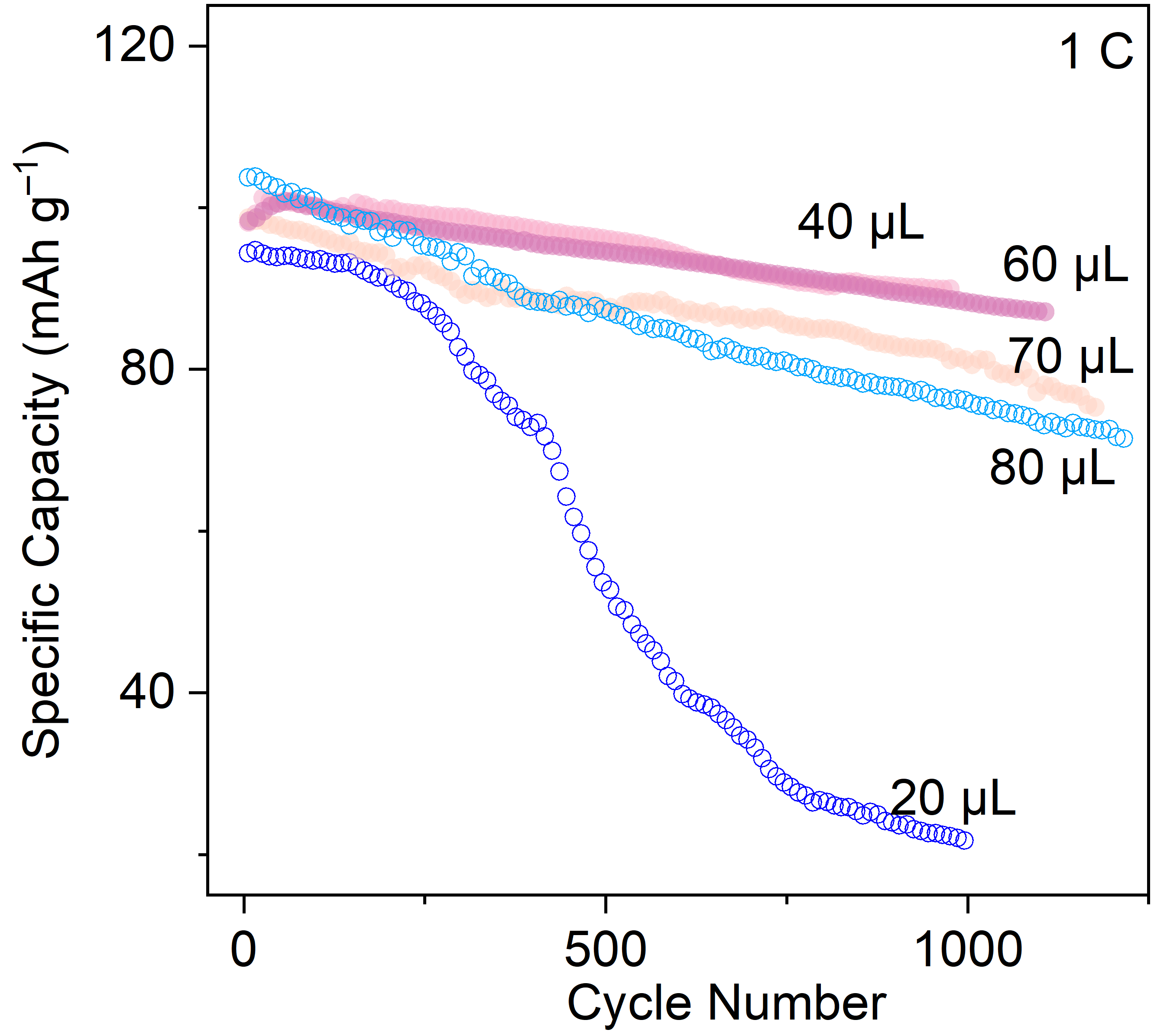


**Fig. S13** Cycling performances of varied amount electrolyte in NVP||Sn-FB QSE||Na cells at 1C.

**Table S3** Comparison between Sn-FB QSE with other reported works

| Sample | CCD | Anode Stability  (0.1 mA cm^−2^) | Specific  Capacity  (1C) | Longest  Cycling  Number | Max Rate  (C) | Reference |
| --- | --- | --- | --- | --- | --- | --- |
| **Sn-FB QSE** | **3.0** | **6000** | **100.8** | **2000** | **15** | **Our Work** |
| BiCl_3_@NZSP | 2.0 | 3000 | 109.5 | 300 | 1 | [S3] |
| BSCPE | 2.0 | 8800 | 82.7 | 1200 | 2 | [S4] |
| UNSCE | / | 1000 | 99.3 | 500 | 10 | [S5] |
| HFP-PC-FEC-Sn | 2.0 | 3000 | ~92 | 1000 | 5 | [S6] |
| PEO/PAM-BC-1,2-DBB | 2.1 | 1200 | 90.3 | 300 | 1 | [S7] |
| SPCE | 2.7 | / | 91.6 | 5000 | 10 | [S8] |


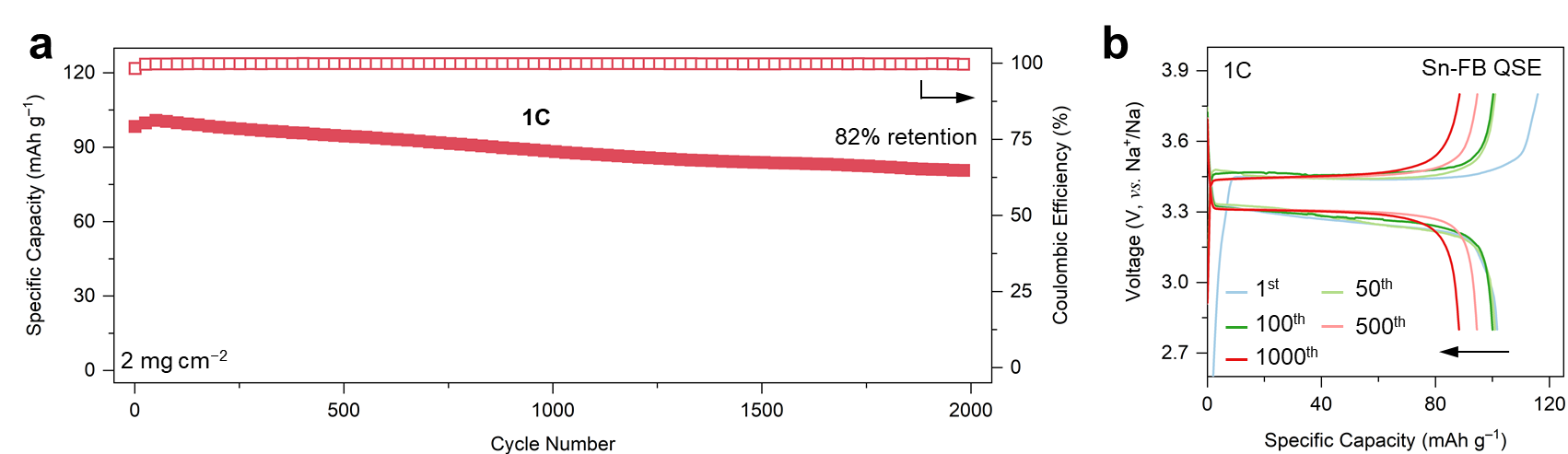


**Fig. S14** Cycling performances in NVP||Sn-FB QSE||Na full cells at (a) 1C and (b) corresponding GCD curves at an interval of 1^st^, 50^th^, 100^th^, 500^th^, and 1000^th^. Points plotted in (a) were taken every 25 cycles and the cell in (a) was all first activated at 0.5C for 5 cycles, which are not shown.


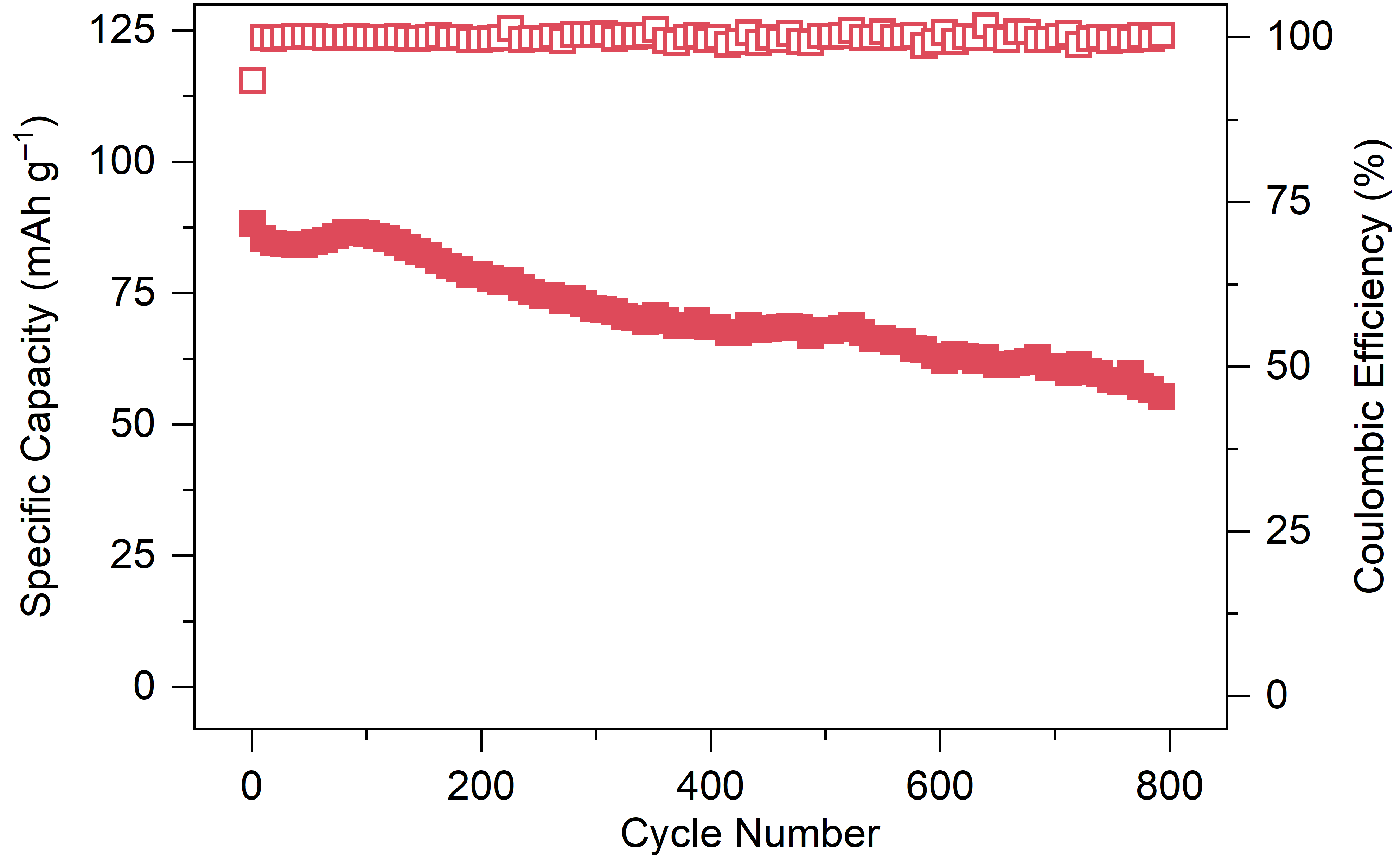


**Fig. S15** Cycling performances in NVP||Sn-FB QSE||Na cells at 5C.


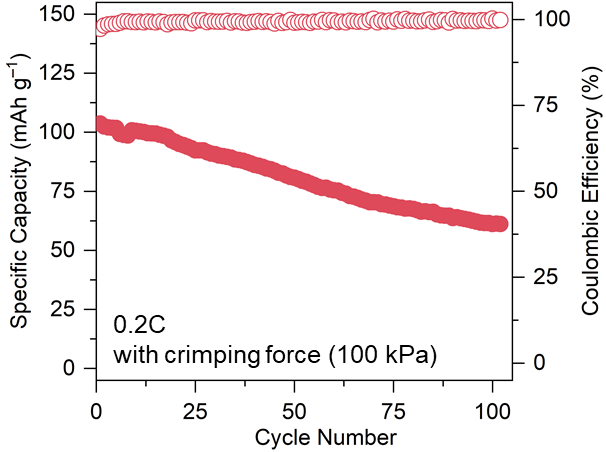


**Fig. S16** Cycling Performance of Sn-FB QSE pouch cell at 0.2C under a minimal external pressure of 100 kPa.


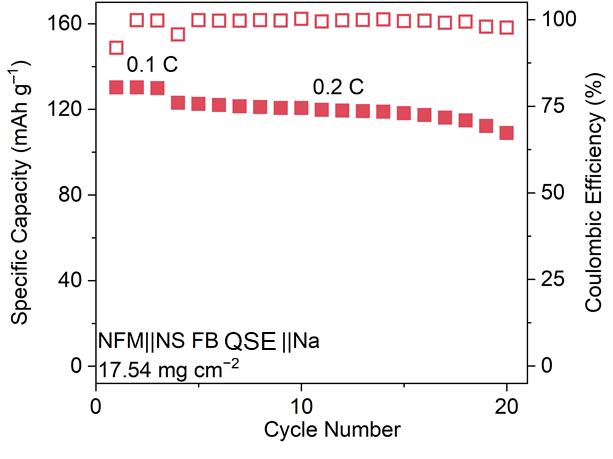


**Fig. S17** Cycling performance of NFM||Sn-FB QSE||Na cell between 2.0 and 4.0V (cathode mass loading: 17.54 mg cm^−2^).


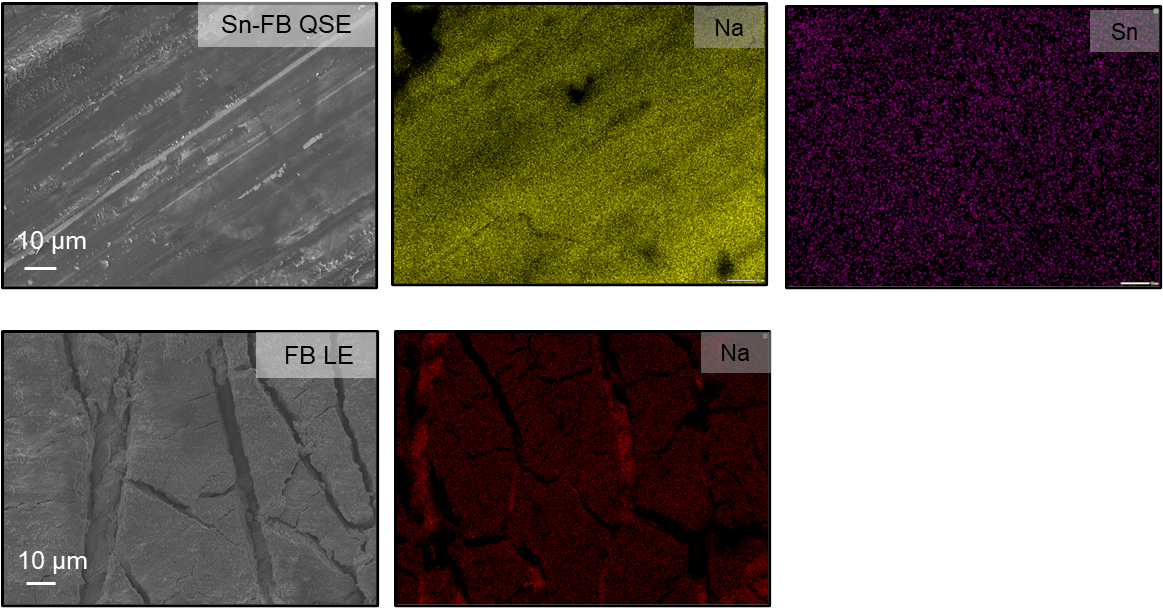


**Fig. S18** SEM images and corresponding elemental mappings of plated Na anode from Sn-FB QSE and FB LE in Na||Na symmetric cells.


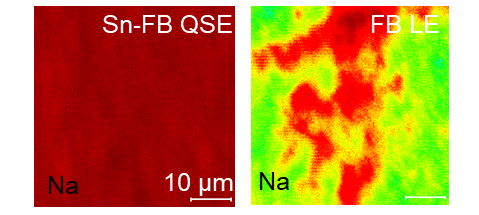


**Fig. S19** 2D rendering visualizations from ToF-SIMS of Na^+^ on Sn-FB QSE and FB LE.


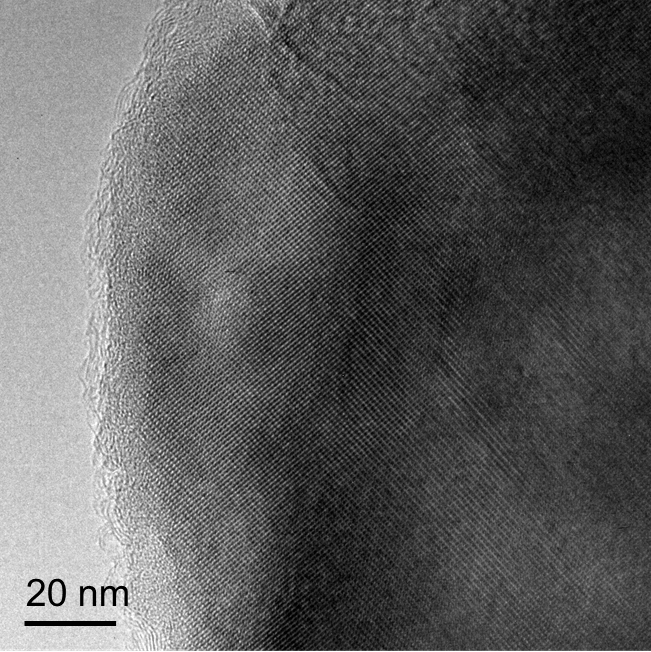


**Fig. S20** High-resolution TEM image of NVP from Sn-FB QSE.


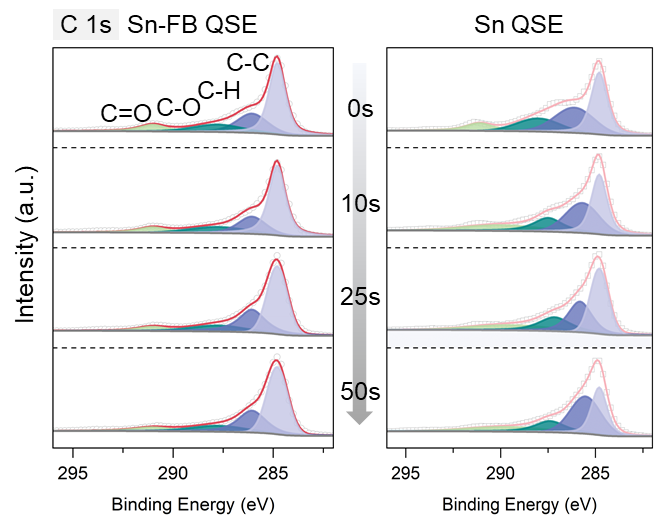


**Fig. S21** In-depth C 1s XPS profiles of Sn-FB QSE and Sn QSE.


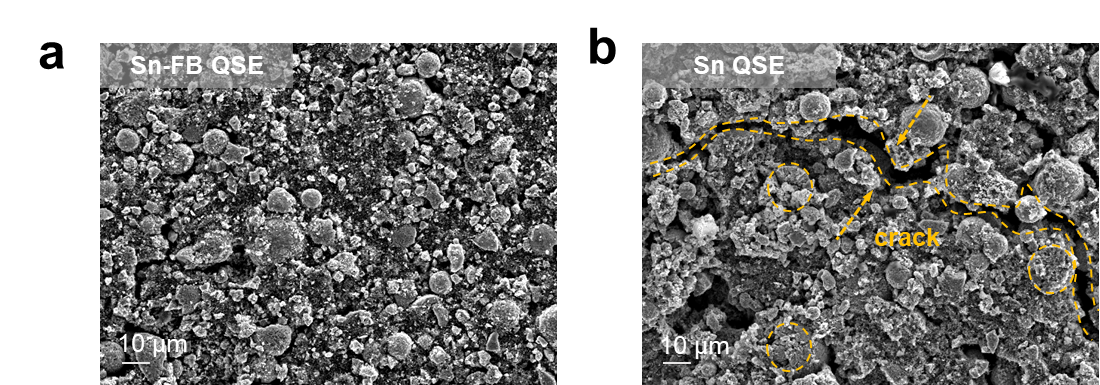


**Fig. S22** SEM images of cycled NVP from (a) Sn-FB QSE and (b) Sn QSE.

#### Supplementary References

[S1] M. J. Frisch, G. W. Trucks, H. B. Schlegel, M. A. Robb, J. R. Cheeseman et al., Gaussian 16 Rev. C.01. Wallingford, CT, (2016).

[S2] J. Zheng, W. Zhang, C. Huang, Z. Shen, X. Wang et al., In-situ polymerization with dual-function electrolyte additive toward future lithium metal batteries. Mater. Today Energy **26**, 100984 (2022). <https://doi.org/10.1016/j.mtener.2022.100984>

[S3] Y. Gu, H. Ma, X. Fan, H. Tao, X. Yang et al., A multifunctional Na_x_Bi/NaCl flexible interface layer for solid-state Na metal batteries. Adv. Funct. Mater. **35**, 2416077 (2025). <https://doi.org/10.1002/adfm.202416077>

[S4] J. Guo, F. Feng, X. Jiang, R. Wang, D. Chu et al., Boosting Selective Na^+^ Migration Kinetics in Structuring Composite Polymer Electrolyte Realizes Ultrastable All-Solid-State Sodium Batteries. Adv. Funct. Mater. **34**, 2313496 (2024). <https://doi.org/10.1002/adfm.202313496>

[S5] L. Zeng, C. Bao, X. Xin, H. Lu, P. Xiong et al., 3D NASICON Ceramic Skeleton Enabled 18 μm-Thick High Performance Bicontinuous-Phase Ultrathin Composite Quasi-Solid-State Electrolyte. Adv. Funct. Mater. **36**, e17736 (2026). <https://doi.org/10.1002/adfm.202517736>

[S6] Z. Yang, H. Jiang, X. Li, X. Liang, J. Wei et al., Fabricating Wide-Temperature-Range Quasi-Solid Sodium Batteries with Fast Ion Transport via Tin Additives. Adv. Funct. Mater. **34**, 2407713 (2024). <https://doi.org/10.1002/adfm.202407713>

[S7] Y. Guo, J. Liu, A. Shao, L. Cheng, J. Tang et al., Coordinated Na^+^ Diffusion and Multiscale Interfacial Engineering of Polymer Electrolyte for Room-Temperature Solid Sodium Metal Batteries. Adv. Energy Mater. **15**, 2405104 (2025). <https://doi.org/10.1002/aenm.202405104>

[S8] C. Su, Y. Qu, N. Hu, L. Wang, Z. Song et al., Rapid Na^+^ Transport Pathway and Stable Interface Design Enabling Ultralong Life Solid-State Sodium Metal Batteries. Angew. Chem. Int. Ed. **64**, e202418959 (2025). <https://doi.org/10.1002/anie.202418959>
